# Distribution and ecotoxicological assessment of trace elements and minerals in Nile Delta sediments using multitechnique analysis

**DOI:** 10.1038/s41598-025-09940-w

**Published:** 2025-07-17

**Authors:** Mohamed A. Hassaan, Marwa R. ElKatory, Wael Abdelwahab, Murat Yılmaz, Ahmed El Nemr

**Affiliations:** 1 National Institute of Oceanography and Fisheries (NIOF), Kayet Bey, Elanfoushy, P.O. 21556, Alexandria, Egypt; 2 Advanced Technology and New Materials Research Institute, SRTA-City, New Borg El-Arab City, Alexandria 21934 Egypt; 3 Geological Sciences Department, National Research Centre, El-Buhouth St., Dokki, Cairo, 12622 Egypt; 4 Bahçe Vocational School, Department of Chemistry and Chemical Processing Technologies, Osmaniye Korkut Ata University, 80000 Osmaniye, Turkey

**Keywords:** Sediment, Mediterranean, FTIR analysis, PSA analysis, XRD analysis, XRF analysis, Environmental chemistry, Environmental monitoring

## Abstract

This study assesses heavy metal contamination, trace elements, and mineral composition in Nile Delta sediments, a region critical for agriculture and ecology. Sediment samples (n = 23) were collected from key urban, agricultural, and industrial zones using a Van Veen grab sampler to capture spatial variability. Analytical techniques included X-ray fluorescence for elemental composition, particle size analysis for grain size distribution, and X-ray diffraction coupled with Fourier-transform infrared spectroscopy for mineral identification. All analyses followed strict QA/QC protocols, including certified reference materials and triplicate measurements. Statistical analyses (Descriptive Statistics, correlation, PCA) were applied to interpret spatial patterns. The elements analyzed included trace metals and oxides of major elements. Pollution indices—Pollution Loading Index (PLI), Geoaccumulation Index (*I*_geo_), Enrichment Factor, Contamination Factor, and Sediment Quality Guidelines—were used to assess contamination levels and ecological risks. Results showed transition metal concentrations ranging from 0.1 to 569.3 mg kg^−1^, with actual cadmium (Cd) concentrations of 1.0–4.3 mg kg^−1^. The contamination degree ranged from 9.47 to 23.10, indicating localized pollution. Sediments exhibited moderate nickel (Ni) contamination but were within safe limits for copper (Cu) and zinc (Zn). PLI values (0.18–1.09) indicated minimal contamination overall, except at sites E2 and G3. Grain size analysis revealed sand (3.56–95.49%) and clay (0.27–12.50%) variability, while mineralogical analysis identified quartz, calcite, aragonite, albite, microcline, and illite as dominant minerals. The study concludes that Nile Delta sediments are generally uncontaminated by heavy metals (supported by Igeo values), though localized contamination hotspots exist. This research provides novel insights into sediment contamination patterns, offering a critical baseline for environmental management.

**Supplementary Information:**

The online version contains supplementary material available at 10.1038/s41598-025-09940-w.

## Introduction

In recent years, considerable concerns have been expressed about the poisoning of aquatic habitats by heavy metals (HMs)^1^. Due to their resistance to natural degradation, heavy metals (HMs) rank among the most enduring pollutants in ecosystems, encompassing water, sediments, and biota. It turns toxic once the degree of irreplaceability is reached. Heavy metals become harmful when they accumulate in soft tissues and are not broken down by the body^2^. Heavy metal accumulation to dangerous levels in certain environmental conditions could harm the ecosystem^3^. Although copper, zinc, iron, and manganese are deemed essential metals due to their critical roles in biological processes, higher concentrations can be dangerous^4–6^. Non-essential metals such as Pb and Cd can lead to intoxication, tissue and cellular damage, decreased fertility, organ dysfunction, and even cell death when they bioaccumulate in tissues. Even in extremely low quantities, these metals are frequently potent toxins^7,8^. Lead and cadmium are listed as hazardous metals in the European Union’s hazardous metal standards, and nickel and chromium have been classified as such by the US Food and Drug Administration^9^. Due to their limited solubility in water, metals are adsorbed and accumulate in bottom sediments^2,10^. Metals deposited in sediments can re-suspend, leading to increased aquatic environment contamination, as sediments serve as both a source and a sink for metals. Because of this feature, the sediments have become an enduring record of the anthropogenic contaminants that have been added. Hence, to fully understand the mechanisms that drive the accumulation and geochemical distribution of HMs in aquatic environments, as well as to provide crucial information for assessing risks to human health, it is imperative to conduct spatial surveys of metal concentrations in sediments and juxtapose these findings with uncontaminated baselines.

Various techniques have been developed to evaluate the ecological concerns associated with HMs. Most of them, meanwhile, like the Geoaccumulation index (*I*_geo_) approach and Enrichment factor (*EF*), are only suitable for the ecological assessment of a single contaminant. Diverse heavy metals frequently coalesce, resulting in cumulative contamination. Sediment Quality Guidelines* (SQG)* were developed to assess the potential effects of pollutant mixtures in sediments^11^.

Egypt’s Mediterranean shoreline extends over 1200 kms from Rafah to El-Salloum^12,13^. Regretfully, substantial pollution flows into most of Egypt’s Mediterranean coastal zones due to many human activities^14,15^. Egypt’s Mediterranean coast is divided into eight governorates. The locations, listed from west to east, are Matruh, Alexandria, Behaira, Kafr El-Sheikh, Damietta, Daqahliya, Port Said, and North Sinai. The large urban populations and nearby agricultural areas substantially drive pollution in coastal waters. The origins of these pollutants were drainage channels discharging directly into the sea, such as "El-Tabia and El-Ummum," the irrigation canals of Mahmudiya and Nubariya, the Rosetta branch of the River Nile, and the coastal lagoons, including Maryut, Idku, Burullus, and Manzala. Large areas of the Nile Delta have been badly impacted by coastal erosion, even with proper protective and mitigating measures being taken into consideration. But most coastal lagoons, or "lakes," are endangered because of excessive sewage discharge from farms, enterprises, and residences^12,15,16^.

The Alexandria Governorate’s primary industries play a significant role in the coastal zone’s metal pollution. These industries include fertilizers, paper, detergents, food processing, textiles, agrochemicals, building materials, pulp, power plants, fibers, and dyestuffs. The average daily industrial effluent amounts to 1–2 million cubic meters of domestic sewage and 30,000–261,000 cubic meters of agricultural waste^12,17,18^. The locations that drain intensively agricultural areas and the outskirts of large towns or industrialized areas, are frequently the only places where heavy metals may have an impact^19^. Once introduced into the ecosystem, heavy metals can affect marine species’ ability to reproductive cycles, photosynthesize, sediment nutrient cycling, cell development, and regeneration^20^. HMs such as lead (Pb) and cadmium (Cd) are considered valuable indicators of pollution resulting from human activities^21^. In children, the primary pathway for lead absorption is oral ingestion of contaminated dust and soil^22^.

Furthermore, Cadmium (Cd) may be present in some phosphate-containing fertilizers, which could be a significant source of dietary Cd absorption for humans. Moreover, after wastewater treatment, sewage sludge contains significant levels of Cd^23^. The term "leachable metal fraction" refers to the proportion of metals that are anthropogenically present in sediment particles. Diluted acids have been used extensively to recover leachable fractions^24–26^.

Organic and inorganic particles come into contact with the coast through various channels, which make up coastal sediments. In environmental studies, particle size analysis is frequently used to identify sediments’ source, deposition, and transit mechanisms. The composition of sediment particle sizes is strongly influenced by the energetic and hydrodynamic conditions of depositional environments, as these size characteristics indicate the unique conditions within each habitat^27,28^. The distribution of particle sizes is affected by various factors, including geography, environmental conditions, and the source of the particles^29^.

Mineralogical analysis reveals that these sediments serve as sensitive environmental indicators, providing valuable insights into regional hydrodynamics, including patterns of sediment transport and deposition^30,31^. Because certain minerals are found in more significant concentrations in the silt and clay fractions, the relationship between silt content and clay mineralogy can occasionally be more direct. Sand fractions, however, include different minerals^32^. Fine-grained marine sediments from fluvial sources are deposited mainly in nearshore areas and on continental margins, with minimal amounts transported to deeper ocean regions^33^. The relative number and composition of the clay minerals are determined by the weathering conditions and their source rocks. On the continental slope and shelf, sediment distribution is primarily shaped by depositional processes, with primary influences from prevailing circulation patterns and the energy conditions affecting clay mineral settling^34^. Comprehending these mechanisms aids in forecasting the transportation routes of contaminants that are selectively regulated inside these fine-grained sediments^35^.

The clay fraction is known to be richer in metal oxides, which are mostly found in the lattices of clay minerals and include Fe_2_O_3_, MgO, and MnO^36^. The highest enrichment of zinc, cadmium, lead, organic matter, and copper is expected to occur in the finest sediment fractions, such as silt and clay, as these clay minerals and oxides are known to be particularly effective adsorbents of trace elements^36^. Because of this exceptional quality, sediments are employed as geo-markers in the aquatic environment to monitor and detect possible sources of pollution^37^. Sediment analyses offer insights into pollution indicators within the ecosystem and are essential for identifying pollutants as they build on the seafloor through diverse chemical constituents. Due to various chemical reactions, sediments can accumulate metals and are typically found in marine aquatic settings^37^.

Furthermore, organic matter is influenced by the properties of the sediment, particularly the particle size, which is separated into labile and refractory fractions^38^. Mineralization causes the labile percentage of organic matter to decrease from shallower to deeper waters, but the refractory portion stays intact. Therefore, a refractory fraction makes up the majority of organic matter^39,40^.

Numerous researchers have analyzed the mineral composition of marine sediments, focusing on the speciation and distribution of clay minerals to understand their sources and depositional patterns^41–43^. The very small particles of clay minerals, often less than two millimetres in diameter, are hydrated aluminum silicates^44^. Even while contemporary spectroscopic methods may examine the sediment’s surface at the molecular level, they are not frequently used in sediment research^45^. Heavy metals in soil and sediments are often analyzed by atomic spectrometry techniques (ICP-OES, AAS)^46–51^. In many mineralogical investigations, the XRD technique is employed. X-ray fluorescence (XRF), X-ray diffraction analysis (XRD), and Scanning Electron Microscopy (SEM)are among the analytical techniques that are frequently employed in mineralogical research^30,31^. These techniques are popular because they are accurate and efficient. The many varieties of clay minerals may be distinguished and the structural changes resulting from chemical changes can be studied thanks to the FTIR technique^52^.

This study investigated the ecological risk and levels of trace metal contamination in sediments by utilizing multiple pollution indices, including the sediment quality guidelines (*SQGs*), Contamination degree (*Cd*), Contamination Factor (*CF*), Geoaccumulation Factor (*Igeo*), the Enrichment Factor (*EF*), and Pollution Load Index (*PLI*). The sources of heavy metals in sediments were identified using Pearson’s correlation matrix and principal component analysis (*PCA*). This study addresses critical knowledge gaps through:

- Determining spatial contamination patterns and discriminating against anthropogenic vs. natural metal sources using XRF analysis across 23 representative sites.
- Quantifying mineralogical controls on HM retention via integrated XRD/FTIR characterization.
- Evaluating ecological risks using updated *SQGs* and multi-index assessment (*PLI*, *I*_geo_, *EF*, *CF*).
- Establishing grain size-mineralogy-contaminant relationships to identify pollution hotspots.
- identified the sources of heavy metals in sediments using Pearson’s correlation matrix and principal component analysis (*PCA*).

By combining modern analytical techniques with spatial and statistical tools, this study offers an integrated framework for understanding the mechanisms driving heavy metal accumulation in one of Egypt’s most ecologically and economically critical coastal zones.

## Materials and methods

### Study area and sampling

The research area (Fig. S1, Software QGIS 3.18; https://www.filehorse.com/download-qgis/61739/) is situated in the central region of Egypt’s Mediterranean coast and spans the Nile Delta coastal area between Abu Qir Bay in the west (31,022′ N—30,018′ E) and Manzala Lagoon outflow in the east (31,017′ N—32,012′ E) (Table S1). This region contains three coastal lagoons: Burullus, Edku, and Manzala. The delta drainage system contributes runoff to these lagoons^18^.

Initially, sediment samples were taken in December 2020 during the winter utilizing a van grab sampler on the Salsabil Research vessel (IMO 8815138 of NIOF). Eight sections were used to gather sediment samples; each section includes three locations-1, 2 and 3, depending on depth-with the exception of section A, which has two locations-the first of which was rocky. Between Port Said and Alexandria, on the northern Mediterranean coast, 23 sites were used to gather surface sediment samples at depths ranging from 10 to 50 m (Fig. S1)^18^. This study investigated the Nile Delta coastal area by dividing it into three sectors -the western (A, B, C), central (D, E, F), and eastern (G, H)- with sediment samples collected across eight sections. The samples were stored in spotless glass vials with wide mouths covered with plastic bags^18^. Three replicates (n = 3) were collected from each selected sampling site. The samples were carefully transported in an icebox to the NIOF Laboratory and stored at 4 °C until they were analyzed. Surface samples taken from the top 0 to 5 cm at each location were subjected to XRD, FTIR, XRF, and PSA analyses.

### Analysis and methodology

The sediment samples were air-dried for seven days at 29 °C in a clean, dust-free environment before being processed with an agate mortar and pestle. After going through a 180 mm screen, the materials were homogenized utilizing a Retsch MM200 ball mill fitted with an agate ball compartment for the fine sand fraction. The ASTM D-7348 standard test technique for loss of ignition (LOI) on solid combustion and the ASTM E-1621 standard guide for elemental analysis via wavelength-dispersive X-ray fluorescence spectrometry is the protocols employed by the Axios PANalytical 2005 Sequential WD XRF Spectrometer at the National Research Center Laboratory. The multi-element synthetic standards were produced by BGS/PANalytical Corporation, and the data was analyzed using the advanced data processing software of the PANalytical Super Q package. Major element data were obtained from samples that were automatically fused with 66% lithium tetraborate and 33% lithium metaborate in an electric furnace at a 1 to 10 g ratio of flux agent. The Ominion/WROXI software’s mathematical methods are used to scan the spectrum. Traces and a few REEs were extracted from a 6-g sample and were automatically pressed by a Herzog pressing machine into a 1.5-g binder wax. The PRO-TRACE program scans and processes the desk spectrum.

The surface sediment samples obtained were subjected to grain-size analysis for PSA. In a 600 mL measuring beaker, a suitable quantity of sediment was evenly distributed for every sample. Subsequently, the sample was analyzed utilizing a Mastersizer 3000 laser grain-size analyzer (Malvern Instruments Ltd. 2017). Conversely, various minerals were identified using FTIR and XRD methods. A suitable quantity of the well-homogenized, dried, and milled silt was measured using a wavenumber between 400 and 4000 cm^−1^ utilizing Fourier Transform Infrared (FT-IR) spectroscopy V-100 VERTEX70 linked to a platinum ATR (model-100). X-ray diffractograms (XRD) with a Cu tube (λ = 1.54060 Å) ranging from 0 to 80° are conducted at 30 kV, 10 mA, using a Bruker 2D Phaser.

### Pollution indices

#### Pollution quantification

Numerous criteria were utilized in the assessment of sediment pollution and ecological risk. Interpretive tools like numerical sediment quality recommendations (*SQGs*) are often used to assess specific contaminants’ ecological risks or biological significance^53,54^. Evaluating if the concentrations of HMs in the sediments of the examined area endanger aquatic life is essential. This assessment employs two categories of sediment quality guidelines (*SQGs*): threshold effect concentrations (*TECs*), encompassing effect range low (*ERL*), threshold effect level (*TEL*), and logistic regression of T20, are utilized to determine concentrations that are unlikely to adversely affect sediment-dwelling organisms, while probable effect concentrations (*PECs*), which include probable effect level (*PEL*), effect range medium (*ERM*), and logistic regression of T50, are employed to identify concentrations that are likely to have detrimental effects on these organisms^55–58^.

The Geoaccumulation index (*I*_*geo*_) for the examined metal was calculated using Eq. (1) to assess the extent of HM contamination.

1 $${I}_{\text{geo }}= {\text{log}}_{2}\left( \frac{{C}_{\text{n}}}{1.5{B}_{\text{n}}}\right)$$

where *B*_*n*_ is the baseline shale value(Müller, 1981) and *C*_*n*_ is the discovered heavy metal content. The seven classes that comprise the geoaccumulation index are: Practically unpolluted sediment was classified as class 0 (*I*_*geo*_ ≤ 0), polluted to a moderate degree was classified as class 1 (0 < *I*_*geo*_ < 1), moderately to heavily polluted as class 2 (1 < *I*_*geo*_ < 2), moderately to heavily polluted as class 3 (2 < *I*_*geo*_ < 3), heavily polluted as class 4 (3 < *I*_*geo*_ < 4), heavily to extremely polluted as class 5 (4 < *I*_*geo*_ < 5), and highly polluted as class 6 (*I*_*geo*_ > 5)^58^. The enrichment factor (*EF*) values for the metals under investigation around the shale average^59^ were interpreted using the proposal put forth by Birth^60^, in Eq. (2).

2 $$EF=\frac{{\left(\frac{\text{X}}{\text{Al}}\right)}_{\text{sediment}}}{{\left(\frac{\text{X}}{\text{Al}}\right)}_{\text{shale}}}$$

where x/Al is the ratio of the elements to Al.

The Pollution Load Index (*PLI*) is a straightforward method of illustrating the extent of sediment damage brought on by metal accumulation. *PLI* was determined using Eq. (3).

3 $$PLI ={({CF}_{1}^{\text{i}}\times {CF}_{2}^{\text{i}}\times {CF}_{3}^{\text{i}}\dots \dots {CF}_{\text{n}}^{\text{i}})}^{1/\text{n}}$$

where *CF* stands for the level of contamination and *n* for the number of metals. Degraded sediments are indicated by *PLI* > 1, baseline values are indicated by *PLI* = 1, and ideal conditions are shown by *PLI* < 1^61^.

Equation (4) is used to calculate the contamination index (*C*_*d*_), which expresses the total effects of all metals and evaluates the relative contamination of each metal separately^62^.

4 $${C}_{\text{d}}=\sum_{\text{i}=1}^{\text{n}}{C}_{\text{fi}}$$

where the *C*_*fi*_ value was determined using Eq. (5):

5 $${C}_{\text{fi}}=\left(\frac{{C}_{\text{Ai}}}{{C}_{\text{Ni}}}\right)-1$$

The metal’s acquired *i* value is *C*_*Ai*_, whereas *C*_*Ni*_ is its maximum allowable *i* value. For the metal, the contamination factor is the *C*_*fi*_ (*N* stands for normative value). The obtained *C*_*d*_ values are classified as low (*C*_*d*_ < 1), medium (*C*_*d*_ = 1–3), and high (*C*_*d*_ > 3).

#### Statistical analysis and Identification of sources of HMs

The study’s statistical analysis, including multivariate and correlation analyses, was carried out using Microsoft 365, Excel, and SPSS Version 19.

## Results and discussion

### Trace elements

Table S2 presents data of quantitative values in the concentration of certain elements, whereas Table S3 presents concentration values for the major elements.

#### Transition metals (Ag, Co, Hf, W, Fe, Ta V, Ni, Mn, Sc, Zr, Cr, Cu, Mo, Y, Nb).

The average V concentrations in the sediments are higher in G2 and G3, where they surpass 116.9 mg kg^–1^, and lower in A3, H3, and E3 (not reaching 31 mg kg^–1^). Other elements, such as Cr, have average values in A3 sediments that are lower (7.7 mg kg^–1^) and higher (142.7 mg kg^–1^) in E1 sediments. In A2 (0.8 mg kg^–1^) sediments, the average concentration of Co is the lowest, whereas in B2 (13.2 mg kg^–1^) sediments, it is the greatest. Ni is the most concentrated in C3 sediments, reaching a value of 64.4 mg kg^–1^. The lowest average amounts of Cu were found in A2, E3, and H3, whereas the greatest concentrations were found upstream in C3, where they were as high as 16.5 mg kg^–1^. The highest concentration (51 mg kg^–1^) was found in the B3 and G3 samples, while the maximum concentration (Mo) was recorded at B3, never surpassing 2.0 mg kg^–1^ for W.

The average concentrations of Zr for every set of samples vary more than expected, as Table S2 illustrates. A3 sediments have the lowest average concentration, while E3 sediments have the greatest average concentration. The sample of E3 sediments had the greatest Zr content (569.3 mg kg^–1^). C3 sediment samples exhibit the highest average concentration of Nb, reaching a maximum of 28 mg kg^–1^. The average concentration of Nb is often low in other stations; the lowest value is seen in H3, where it is less than 0.1 mg kg^–1^.

**Fig. 1:**
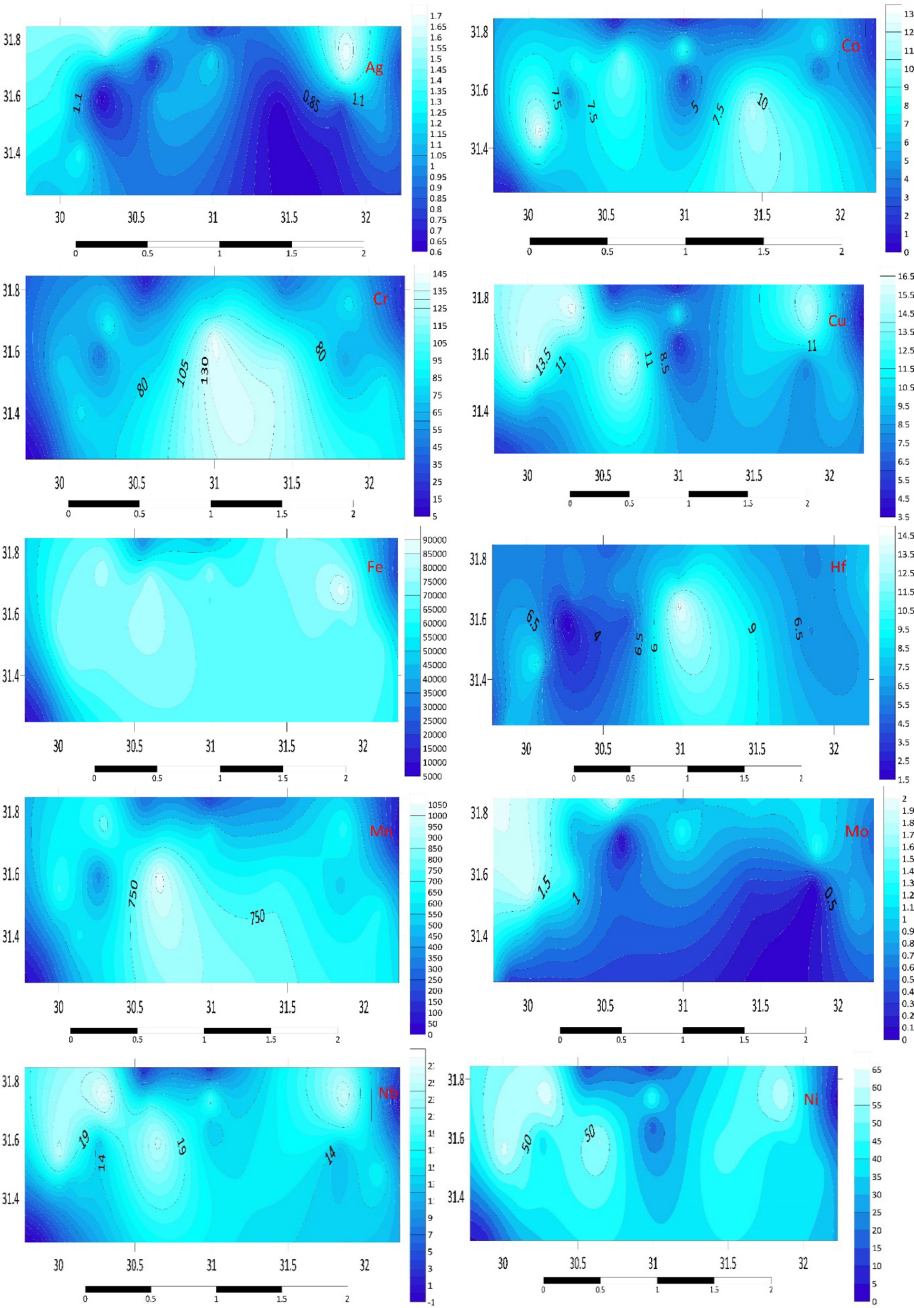

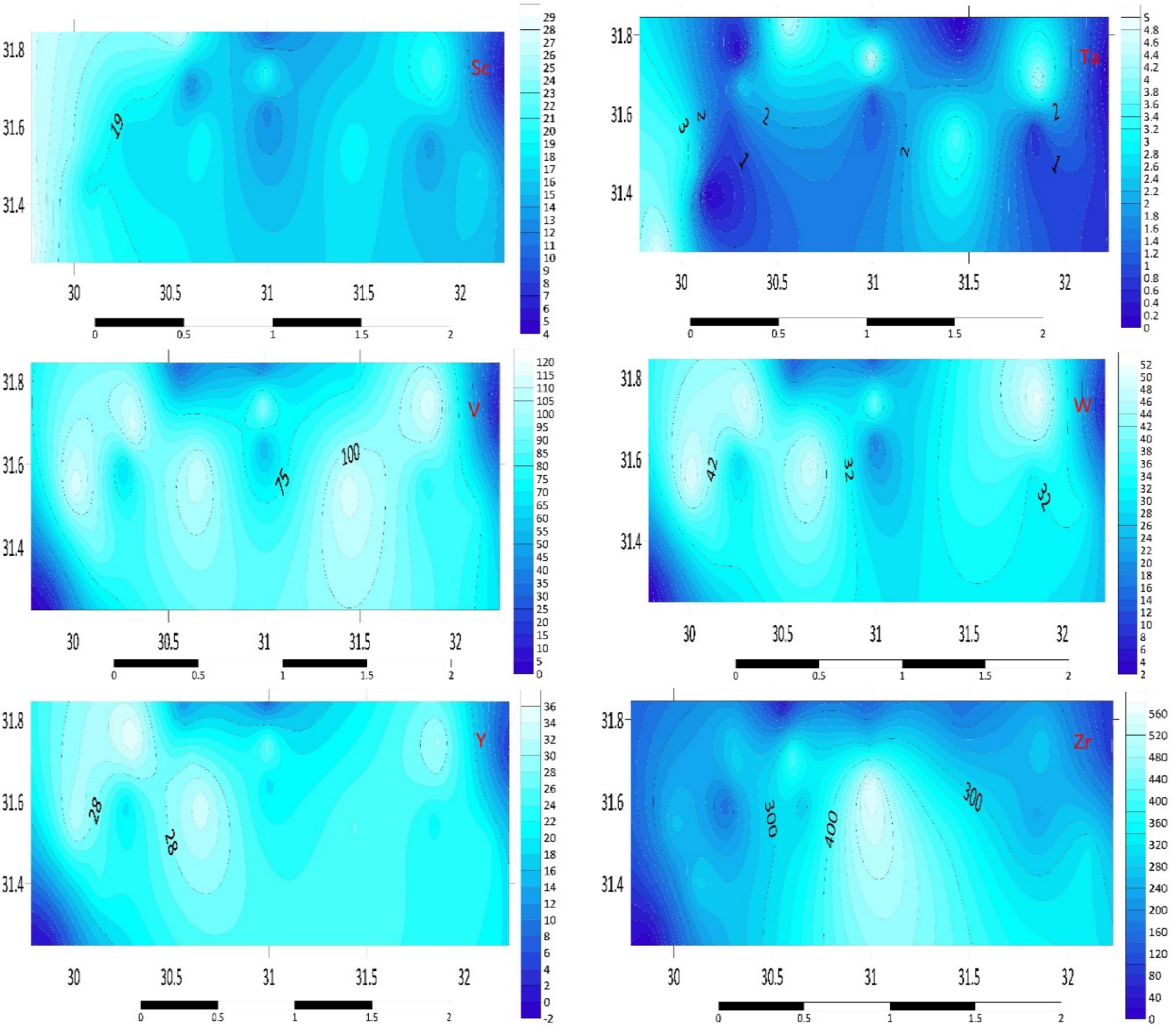
Distribution maps of transition metals (Ag, Co, Cr, Cu, Fe, Hf, Mn, Mo, Nb, Ni, Sc, Ta, V, W, Y, and Zr) in the investigated area.

Furthermore, the A2 sediments exhibited the greatest average content of Ta, measuring (4.9 mg kg^–1^). Figure 1 illustrates the distribution map of transition metals in the investigated area. The elevated concentrations of V, Cr, and Ni in certain stations (e.g., G2, G3, C3) suggest potential contributions from industrial activities, such as metallurgy and chemical manufacturing, as well as natural geological sources. The spatial variability of Cu and Mo concentrations highlights the influence of agricultural runoff, mainly from using fertilizers and pesticides, which are common in the Nile Delta. The high Zr and Nb concentrations in E3 and C3 sediments may be linked to erosion of zircon-rich minerals from upstream areas, reflecting the geological composition of the Nile River basin.

#### Post-transition metals (Tl, Bi, Cd, Ga, Pb, Sn, Zn, Al)

**Fig. 2:**
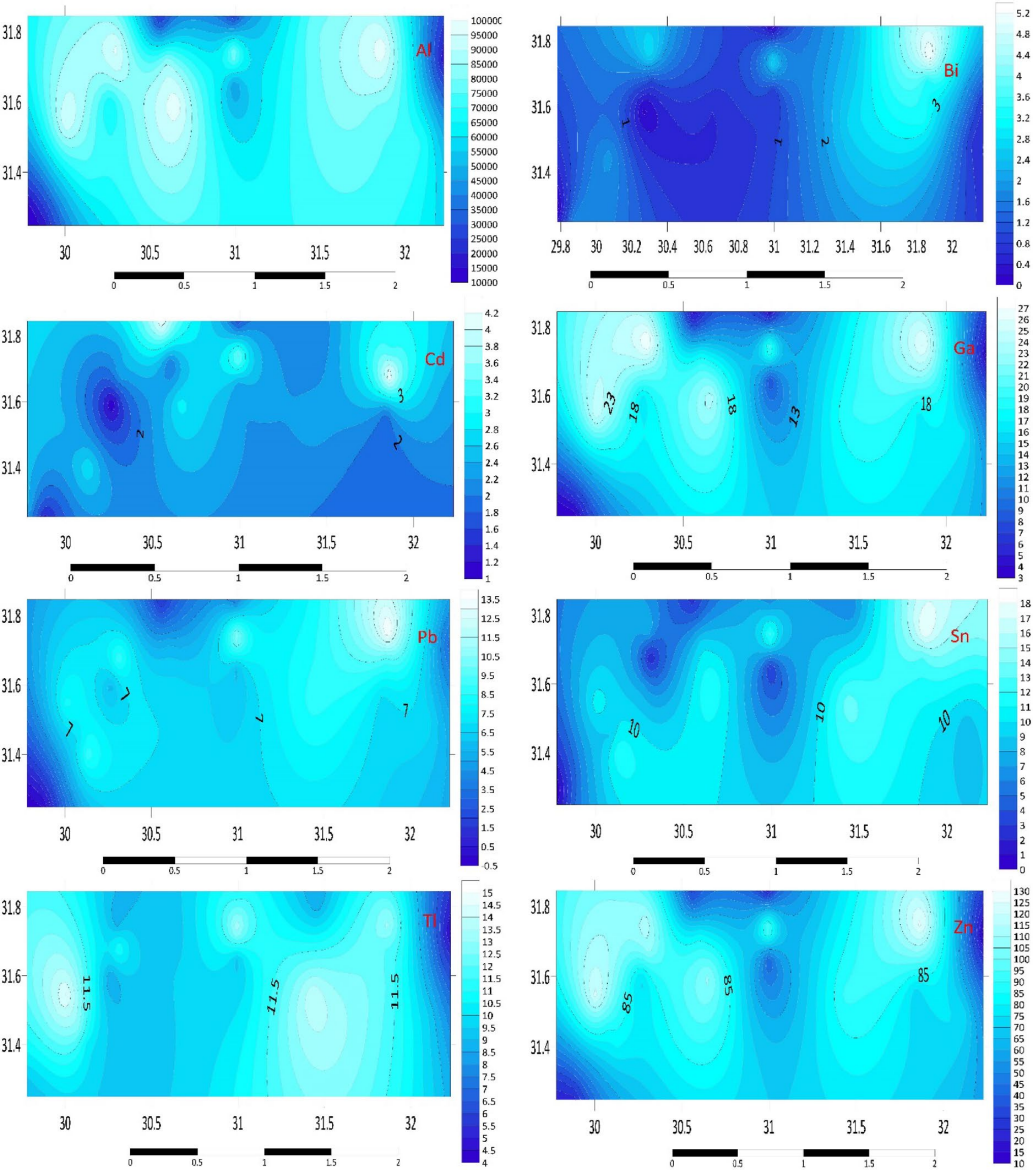
Distribution Map of post-transition metals (Al, Bi, Cd, Ga, Pb, Sn, Tl, Zn) in the investigated area.

The average Tl concentrations in the sediments under study exhibit significant fluctuations, as seen in Table S2. Sediments from E2 and B3 had the greatest average amounts, which didn’t exceed 15 mg kg^–1^. The greatest average concentration of Bi, at 5.3 mg kg^–1^, is shown by the G3 station. The B3 and G3 river bed sediments showed the largest increase in Zn concentration (122.4 & 128.8 mg kg^–1^). D1 sediments had the greatest average Al content (98,557.9 mg kg^–1^). C3 has the greatest average Ga values (27.6 mg kg^–1^). Figure 2 illustrates the distribution map of post-transition metals in the investigated area. The elevated levels of Zn, Pb, and Cd in B3 and G3 sediments are likely associated with industrial discharges and urban runoff, as these metals are commonly used in batteries, paints, and alloys^12,23–26^. The high Al concentrations in D1 sediments may be attributed to the natural weathering of aluminosilicate minerals and contributions from industrial activities such as aluminum processing.

#### Alkali/alkaline earth (Rb, Cs, Sr, Ba)

**Fig. 3:**
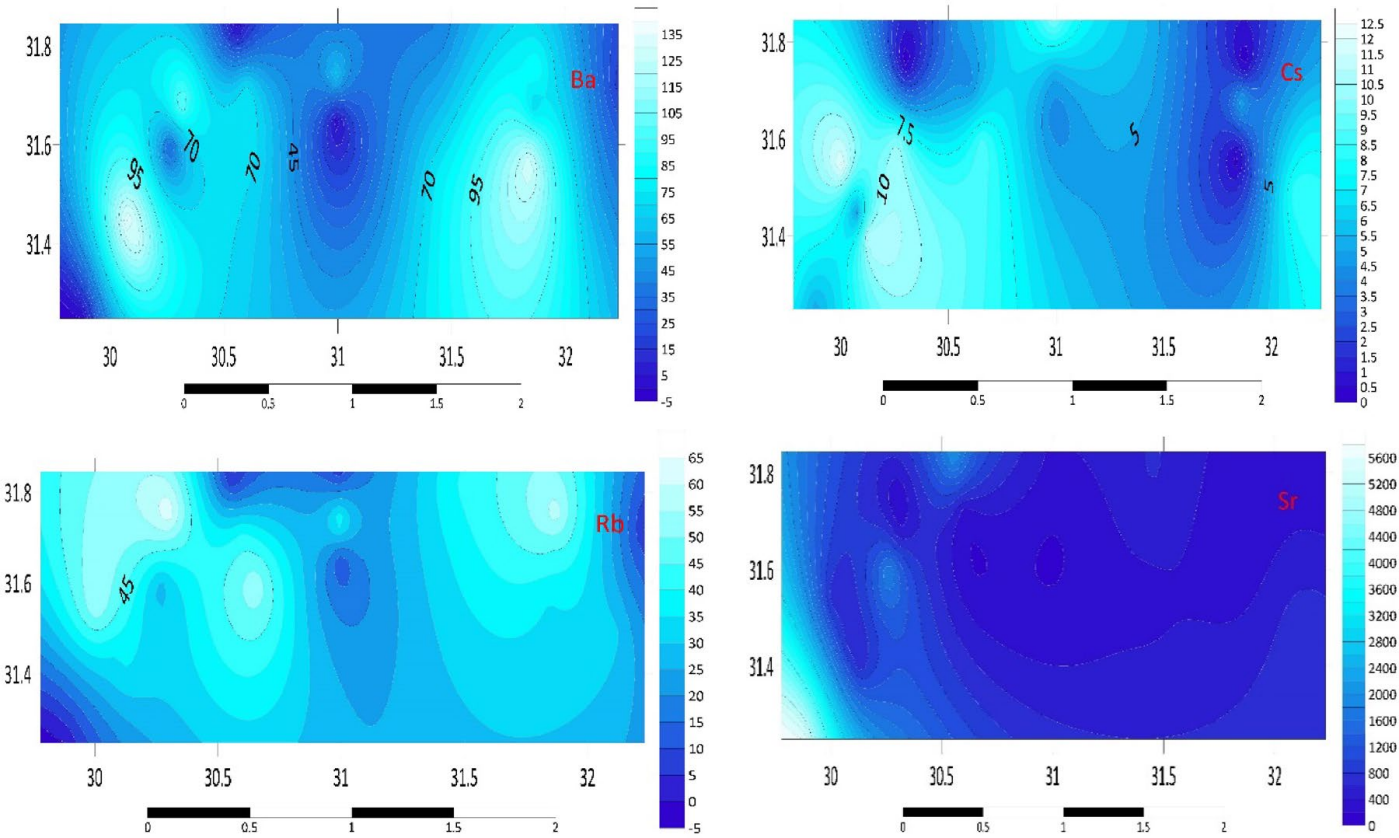
Distribution Map of alkali/alkaline earth (Ba, Cs, Rb, Sr) in the investigated area.

According to the findings from this study, Rb is trending similarly, with the lowest average concentrations found in the D3, E1, E3, and H3 sediments (3, 10.2, 6.1, and 4.7 mg kg^−1^, respectively). Sediments from C3 and G3 showed a significant increase, measuring 64.5 and 57.7 mg kg^–1^, respectively. Regarding Cs, the C2 station has the lowest quantities (4 mg kg^−1^) and the greatest values in every other station. The B3 sample exhibited the highest concentration of Cs, at 12.2 mg kg^–1^. The sediment samples from the Mediterranean region appear to be particularly rich in Sr, particularly in A2, A3, and D3, where concentrations are 5592.7, 5566.7, and 2029.8 mg kg^–1^, respectively. Conversely, the average Ba concentration in C1, E3, and H3 is lower at 35.6 and 17.3 mg kg^–1^. The B1 and B2 sediments had the greatest average concentrations of Ba, measuring more than 100.0 mg kg^−1^ with an average of 126.1 and 136.4 mg kg^–1^, respectively. Figure 3 illustrates the distribution map of the alkali/alkaline earth in the investigated area. The high Sr concentrations in A2, A3, and D3 sediments are consistent with the presence of carbonate-rich deposits, which are characteristic of the Mediterranean region. The spatial distribution of Ba and Rb suggests a combination of natural geological processes and anthropogenic inputs, such as the use of barite in drilling fluids and Rb-containing fertilizers.

#### Metalloids (As, Ge, Sb, Te)

**Fig. 4:**
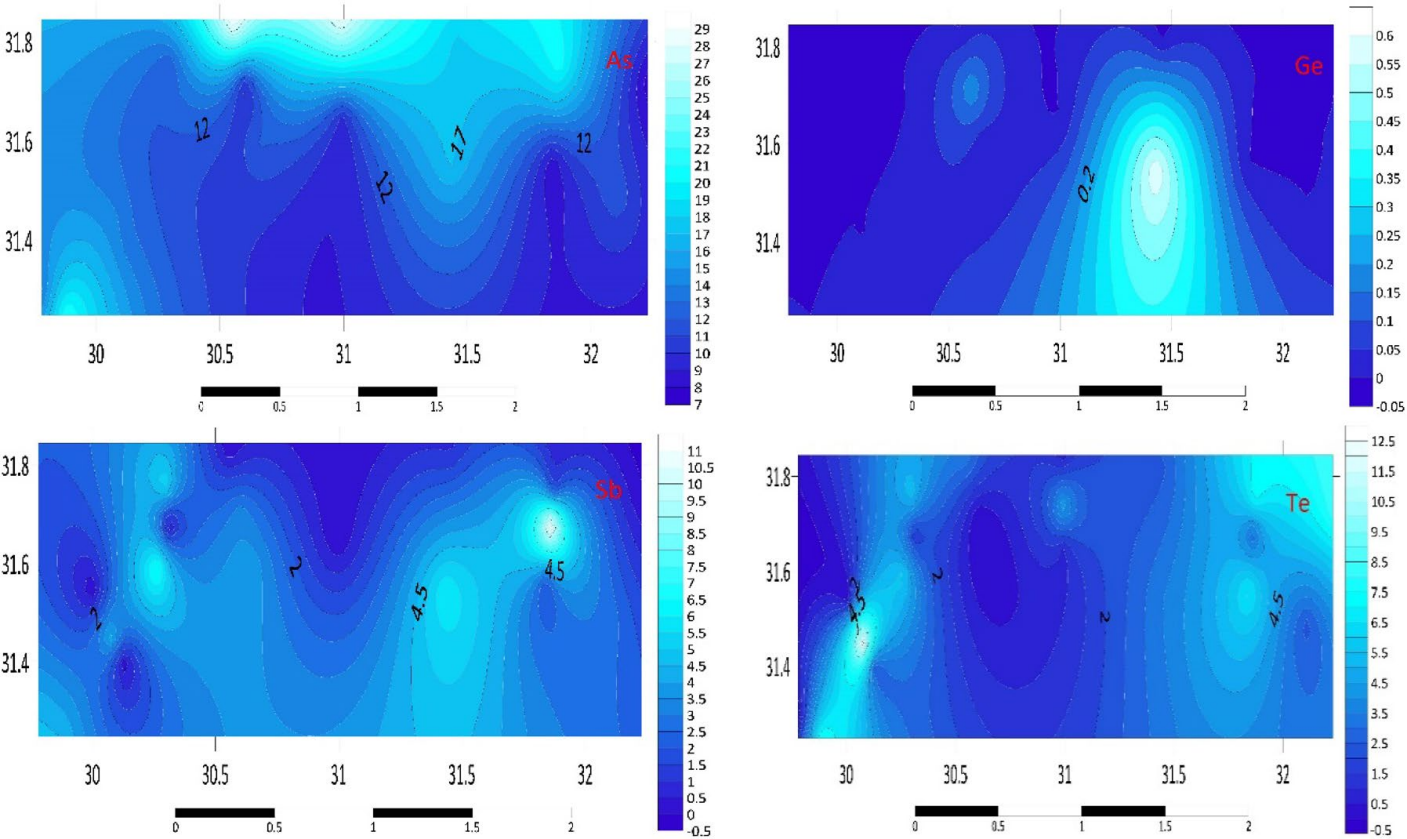
Distribution Map of metalloids (As, Ge, Sb, Te) in the investigated area.

The D3 sample has the greatest concentration of As (29.3 mg kg^–1^) among the metalloids under investigation. With levels as low as 7.4 mg kg^–1^, H3 sediments have the lowest average concentrations. While the highest concentration of Sb was found in G2 sediments, where it reached 11.6 mg kg^–1^, low average amounts were found for Sb (0.1 mg kg^–1^ at the H3 station). The highest average concentrations of Ge were found in F1 stations (not surpassing 0.6 mg kg^–1^) and were nearly absent in all other stations. The B2 sample has a higher average maximum concentration of Te (12.9 mg kg^–1^). In C2 sediments, the average concentration is the lowest, averaging 1.3 mg kg^–1^. The distribution map of metalloids in the studied area is displayed in Fig. 4. The elevated As levels in D3 sediments may be linked to the use of arsenic-based pesticides and herbicides in agriculture, as well as natural geogenic sources. The presence of Sb and Te in G2 and B2 sediments could indicate contributions from mining activities and electronic waste disposal.

#### Reactive nonmetals (Br, I, Se)

The average concentration of Br is greater in A3, reaching 79.7 mg kg^–1^. Conversely, Se concentrations are higher-they reach 3.7 mg kg^–1^ in A2 sediments. A3 sediments have an average I concentration of 75.9 mg kg^–1^, higher than the other sediment samples. The distribution map of reactive nonmetals in the studied area is displayed in Fig. S2. The high Br and I concentrations in A3 sediments may reflect inputs from seawater intrusion and organic matter decomposition, which are common in the Nile Delta’s coastal areas. The elevated Se levels in A2 sediments could be associated with agricultural practices, as selenium is often used in fertilizers and animal feed supplements.

#### Rare earth elements (actinides U and Th)

The U average concentration is nearly nonexistent in the other stations and reaches 0.7 mg kg^–1^ at the G3 station. However, Th concentrations are higher in A2 sediments, reaching 79.1 mg kg^–1^ upstream. Fig. S3 shows the distribution map of rare earth elements in the investigated area. U and Th in G3 and A2 sediments suggest contributions from phosphate fertilizers and natural radioactive minerals.

#### Rare earth element Lanthanides (Ce, La, Nd, Sm)

In C3, the average concentration is greater at 34.3 mg kg^–1^. However, Nd concentrations in C3 sediments are greater, reaching 31.4 mg kg^–1^. Cerium (Ce) has a greater average concentration in C3 sediments, with the greatest value being 65.4 mg kg^–1^. The highest concentration of Sm (17.9 mg kg^–1^) is found in C2 sediments, which have the highest average concentration. The distribution map of the rare earth element Lanthanides in the studied area is displayed in Fig. S4. The high Ce, La, and Nd concentrations in C3 sediments may be linked to the erosion of rare earth element-bearing minerals from upstream areas.

### Major elements

The spatial variations of major elements are shown in Fig. S5 and Table S3. Most stations show higher concentrations of SiO_2_, Al_2_O_3,_ and Fe_2_O_3_, except in A2, A3, and D3 whereas higher concentrations of CaO are observed in these stations.

The major elements of SiO_2_, Al_2_O_3,_ and Fe_2_O_3_ (average 41.06 ± 14.19, 12.37 ± 5.57, and 8.66 ± 3.19%, respectively) are highly enriched for the studied stations’ samples. The A2, A3, and D3 samples are highly enhanced in CaO (average 12.57 ± 12.98) (Fig. S5, Table S3). The loss of ignition (*LOI*) values were documented, averaging approximately 40.18% for A3 and 8.85% for E1, with an overall average of 16.33 ± 9.55% for the entire area. The enrichment of SiO_2_, Al_2_O_3_, and Fe_2_O_3_ in most stations reflects the dominance of silicate and oxide minerals in the Nile Delta sediments. The high CaO concentrations in A2, A3, and D3 sediments are consistent with the presence of carbonate minerals, which are abundant in the Mediterranean region.

### Mineralogy

**Fig. 5:**
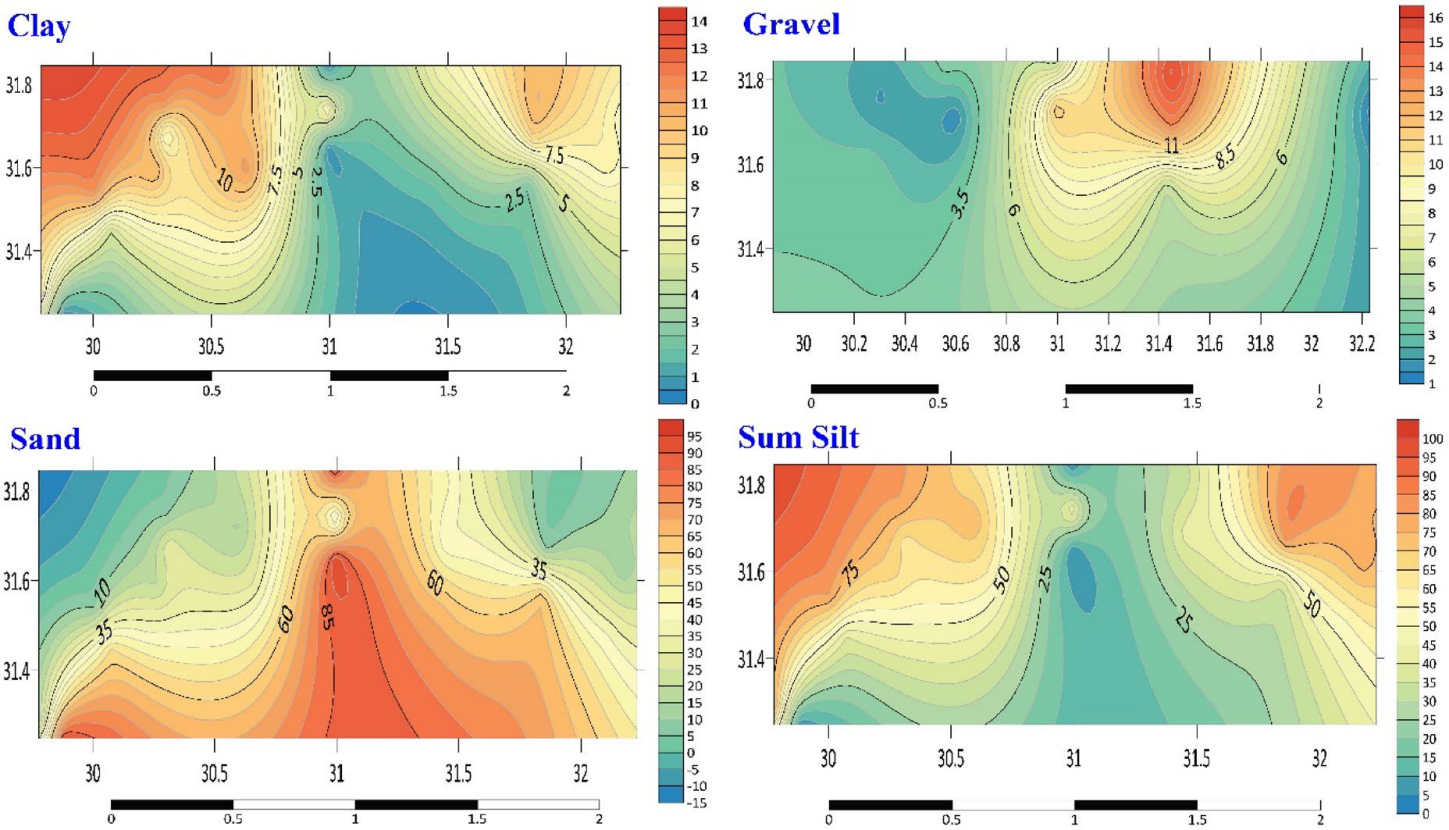
Distribution map of minerals (Clay, Gravel, Sand and Sum Silt) in the investigated area.

In general, there were significant differences in the percentages of clay and sand in the stations under study compared to the findings of the wet PSA analysis. As stated by the results^18^, the percentage of sand varied from 3.56% at station G3 to 95.49% at station E1 (Table S4, Fig. 5). Conversely, the percentage of clay varied between 0.27% at station E3 and 12.50% at station D3^18^.

**Fig. 6:**
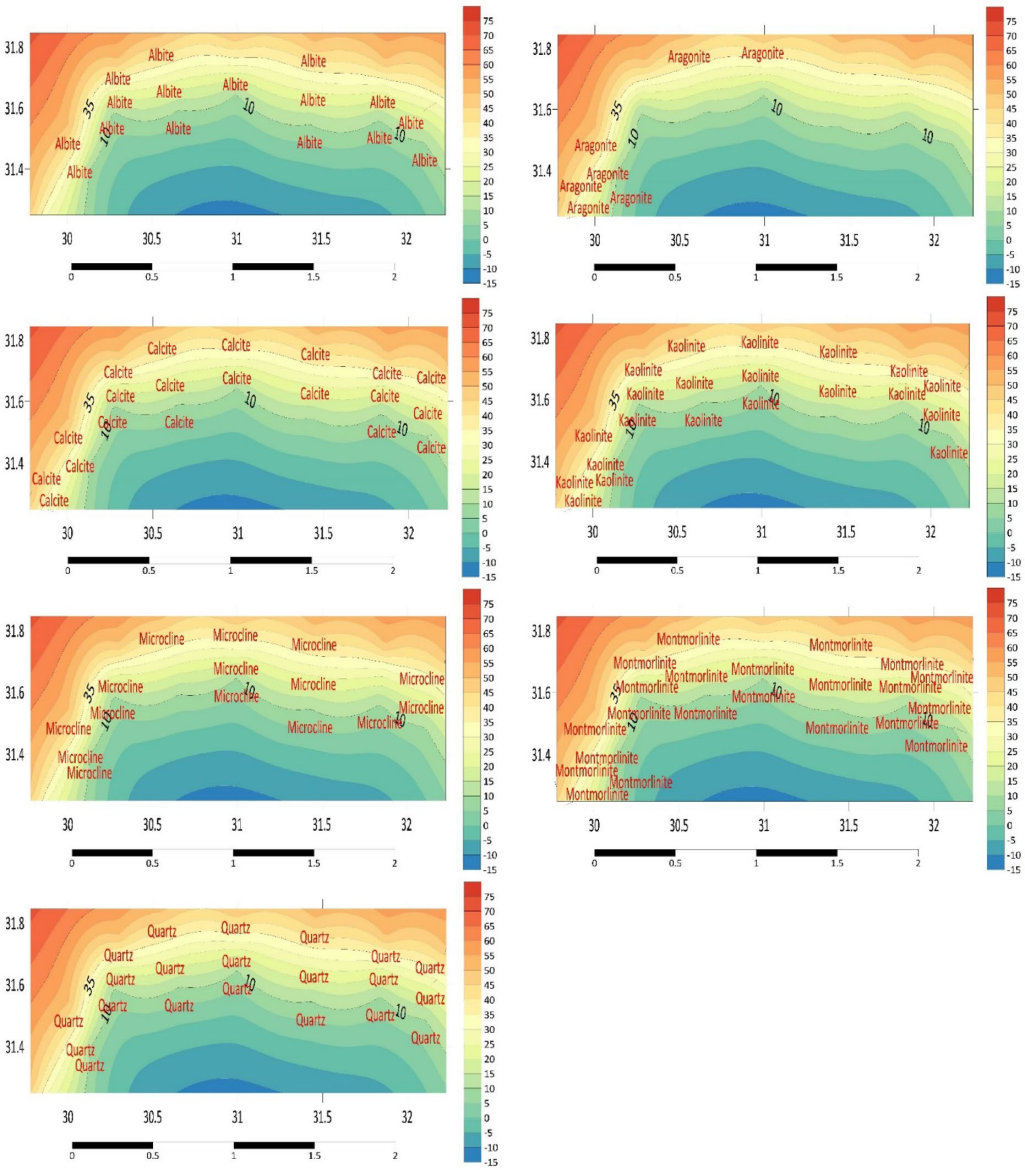
The results of main minerals distribution by FTIR spectra.

The primary mineral distribution results according to FTIR spectra are displayed (Fig. 6). The most prevalent clay minerals are montmorillonite and kaolinite^18^. One silicate mineral that is widely distributed is quartz (Table S3). The presence of quartz provides information about the beginnings of pottery making; kaolin was used elsewhere, while quartz was used in Piedmont to coat covered pottery^18,63^. If quartz is present, it can be determined if the samples originate from the Piedmont. It is impossible to exclude the Piedmont as the source of discoveries containing quartz inclusions^63^. Nonetheless, the preponderance of quartz found in the study’s FTIR analyses would suggest that these samples share a common ancestor^18^.

The synthesis of Ca-silicates or Ca-Al-silicates, including gehlenite (CaAl_2_SiO_7_), diopside (CaMgSi_2_O_6_), and anorthite (CaAl_2_Si_2_O_8_), transpires following the generation of calcite at temperatures above 800 °C, at which point the development of CaO intensifies^18,33,63^. The carbonate component comprises aragonite and calcite and includes both recent and recent biogenic detritus^33^.

**Fig. 7:**
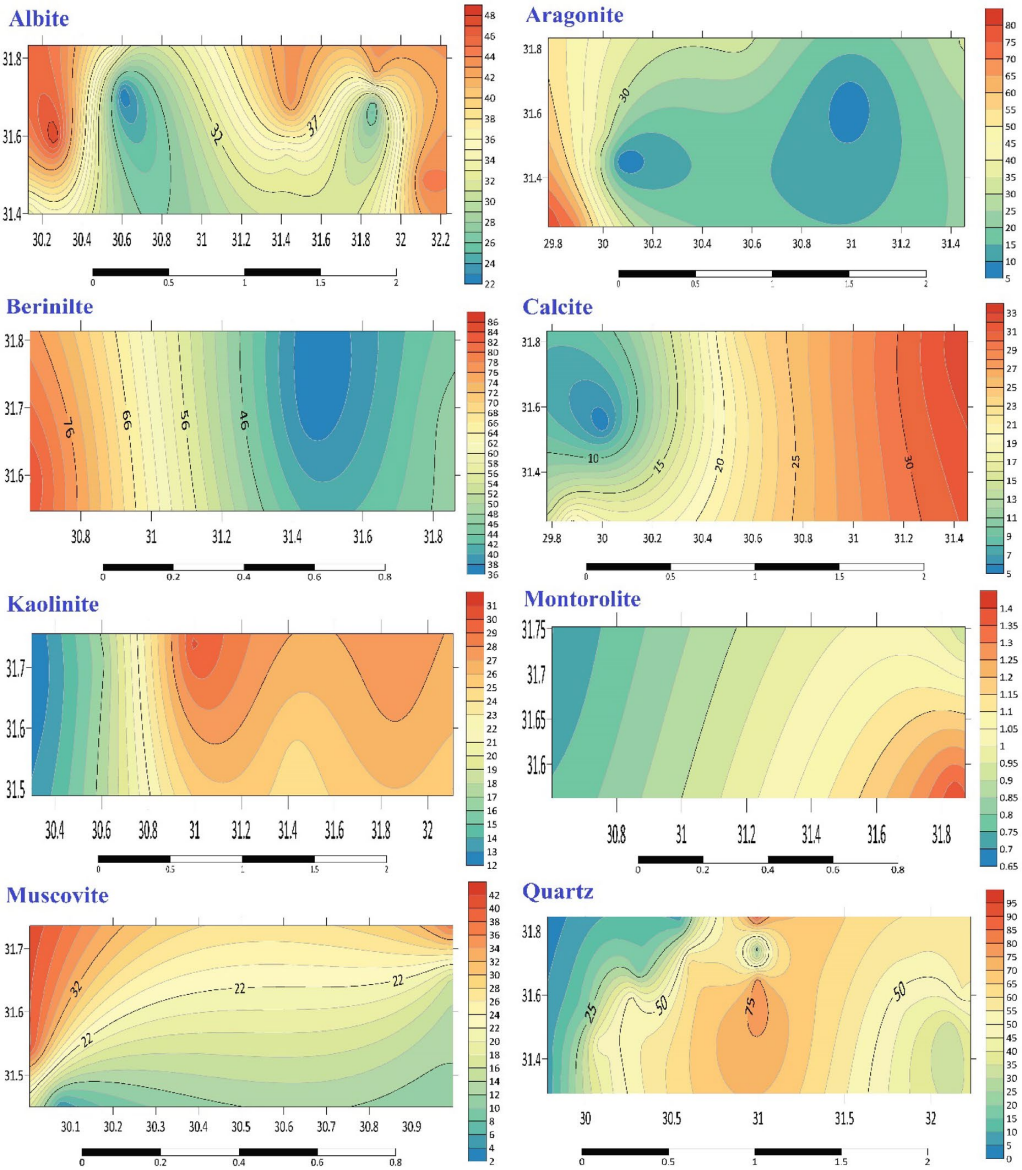
The main minerals distribution by XRD spectra.

The primary mineral distribution data obtained from XRD spectra is provided in (Fig. 7). The mineral composition of the whole sediment sample was ascertained using a diffractogram produced by XRD analysis (Table S6)^18^. The minerals quartz (6.7–91.4%), aragonite (4.9–77%), and calcite (5.2–33%) make up the majority of the sediments (Table S6)^18^. It is also possible to identify feldspar minerals such as illite (0–26.4%), microcline (12.1–30.2%), and albite (21.7–48.1%)^18^. Muscovite, present in concentrations ranging from 3.7 to 37.8%, was identified at sampling stations B2, B3, E1, and E2. In sediment samples such as C2, C3, D2, G1, and G3, component minerals such as montmorillonite (0.7–1.4%) and kaolinite (4.4%) are also found (Table S6, Fig. 7)^18^. In station D1, the mineral belittle is the most prevalent at 84.2%^18^. The crystal structure of beryllium is identical to that of quartz, exhibiting *α*-quartz at low temperatures and *β*-quartz at high temperatures.

Out of all the sediment samples, quartz exhibits the strongest peak, followed by aragonite, albite, calcite, muscovite, illite, and berlinite^18^. Quartz can be found in the majority of study samples. The sample of A2^18^ has the lowest proportion of quartz (6.7%), whereas the sample of E3 has the highest (91.4%) (Fig. 7 and Table S6).

Fine-grained sediments (silt and clay) are deposited at nearshore stations, whereas the distribution of silt and sand grains is affected by moderate to strong currents and wave activity. As a result, sediments composed of silt and sand are displaced from the coastal zone toward offshore areas^64^. Frihy et al.^64^ have documented comparable grain sorting tendencies in the Nile Delta in Egypt. XRD investigations indicate that lower-density minerals, such as quartz, are transported to offshore sediments, where they are deposited in significant quantities, reaching 91.26% at station E3. These fine sediments, which include clay and silt particles smaller than 63 μm, are classified as the most geochemically active components in the sediment (Table S2). Because of this feature, this fraction can be used to assess the possible pollution level in the sediment (Table S2). Fine-grain sediments are an important metal repository and a record of the temporal variations in pollution due to their high adsorption capacity. So, historical reconstruction can make use of them. According to De Groot^65^, this sediment feature can control how much of these chemicals and minerals are in the water column. An essential part of the physicochemical and biological dynamics of metals in marine aquatic ecosystems is also played by marine sediment. It is important to note that the mineralogical data in Tables S5 and S6 point to sediment transported to the delta mainly by the Nile serving as the primary source for the deposits accumulating at the study locations. While illite often decreases, montomonrlite normally increases with an increased proportion of silt fraction. Illite appears to correlate with grain size in the study area, suggesting that it is most likely associated with the transport mechanism that frequently occurs in sand-rich deposits like those at stations C1 and C2. Table S4 shows that somewhat smaller percentages of illite occur in clay-rich deposits.

Numerous minerals in a range of quantities are confirmed to be present by the sediment’s chemical analysis. FTIR and XRD data verified that quartz, aragonite, montomonrlite, calcite, albite, microcline, and muscovite comprised the majority of the sediment locations under study. PSA corroborated the observation that the sediments ranged between silty sand, somewhat gravelly muck, and slightly gravelly sand. Analytical results suggest an increased variance in mixed sediments deposited by wind, waves, and longshore currents. The observation that sediments with a high clay mineral content can retain more pesticide residues than sandy-clay or sandy-silt sediments indicates that the concentration of contaminants in sediments is largely influenced by their capacity to adsorb these pollutants^66^. Table S3 shows that locations with sand-filled sediments may have low concentrations of contaminants such as HMs and pesticides. Moreover, the variation in contaminant concentrations within sediments of differing properties is significantly influenced by the diffusion of contaminants through the pores of the sedimentary layers^66^. Since most of the material was composed of sand, silt, mud, and quartz, most stations may have contained kaolinite, calcite, quartz, and muscovite. Due to this property, sediment can be a suitable indicator for tracking metal pollution.

### Assessment of sediment pollution indices

A commonly utilized metric for assessing trace metal pollution in the sediments examined in this study is the geoaccumulation index (*I*_*geo*_), which was “initially introduced by Muller^67^. Metal contamination in sediments is analyzed and characterized using the *I*_*geo*_^67^. A summary of the calculated *I*_*geo*_ for the sediments may be found in Table 1 and Fig. S6. Tucker’s scale, which indicates that the mean *I*_*geo*_ of these elements is less than zero (*I*_*geo*_ < 0), suggests that heavy metal contamination of the Egyptian Mediterranean Sea has not been substantial overall, except for Cd, which is moderately to heavily polluted in all studied stations.

**Table 1:** The geoaccumulation index (*I*_geo_) in marine sediments.

	As	Cd	Co	Cr	Cu	Mn	Ni	Pb	Zn	Fe	Al
A2	0.13	1.53	− 5.15	− 3.43	− 3.64	− 3.94	− 4.16	0.00	− 2.67	− 2.39	− 2.78
A3	− 0.51	2.47	− 4.15	− 4.13	− 3.59	− 4.71	− 4.06	0.00	− 2.43	− 2.81	− 3.43
B1	− 0.54	2.64	− 1.87	− 0.69	− 2.94	− 1.05	− 1.20	− 1.75	− 0.82	− 0.05	− 0.72
B2	− 0.46	2.42	− 1.11	− 0.90	− 2.83	− 1.20	− 1.16	− 2.10	− 0.74	0.02	− 0.65
B3	− 0.52	2.42	− 1.48	− 0.87	− 2.09	− 0.82	− 0.72	− 1.87	− 0.22	0.03	− 0.33
C1	− 0.74	1.15	− 2.60	− 1.61	− 2.68	− 1.86	− 1.46	− 2.42	− 0.98	− 0.02	− 0.92
C2	− 0.61	2.35	− 1.71	− 0.56	− 2.30	− 0.99	− 0.84	− 1.72	− 0.39	0.08	− 0.42
C3	− 0.69	2.35	− 2.07	− 0.93	− 2.03	− 0.67	− 0.66	− 2.12	− 0.34	0.13	− 0.30
D1	− 0.68	2.69	− 1.68	− 0.84	− 2.07	− 0.29	− 0.86	− 2.16	− 0.41	0.11	− 0.28
D2	− 1.02	2.08	− 1.48	− 1.13	− 2.77	− 1.06	− 1.33	− 2.42	− 0.78	0.15	− 0.49
D3	0.59	3.22	− 4.98	− 3.37	− 3.70	− 2.10	− 3.05	− 4.32	− 2.16	− 1.10	− 2.39
E1	− 1.02	2.29	− 3.76	0.08	− 3.81	− 1.05	− 2.68	− 2.37	− 1.90	− 0.14	− 1.45
E2	− 0.04	3.00	− 1.57	− 0.62	− 2.54	− 1.04	− 1.06	− 1.45	− 0.48	0.07	− 0.52
E3	0.56	1.83	− 8.15	− 0.99	− 4.08	− 3.23	− 4.14	− 3.02	− 2.88	− 0.78	− 2.31
F1	− 0.19	2.35	− 1.33	− 0.11	− 2.74	− 0.80	− 1.13	− 1.79	− 0.65	− 0.10	− 0.67
F2	− 0.16	2.26	− 1.97	− 0.66	− 2.73	− 1.18	− 1.25	− 1.82	− 0.73	− 0.04	− 0.65
F3	− 0.12	2.15	− 3.15	− 1.59	− 2.71	− 1.69	− 1.39	− 1.85	− 0.82	0.02	− 0.62
G1	− 1.23	2.08	− 1.89	− 1.12	− 3.13	− 1.28	− 1.52	− 2.14	− 0.98	− 0.11	− 0.66
G2	− 0.18	3.26	− 3.03	− 0.93	− 2.32	− 1.03	− 0.92	− 1.67	− 0.41	0.35	− 0.39
G3	0.08	2.83	− 1.89	− 0.66	− 2.16	− 0.91	− 0.77	− 1.12	− 0.15	0.02	− 0.28
H1	− 0.73	2.31	− 2.28	− 0.64	− 2.75	− 0.96	− 1.41	− 2.15	− 0.93	− 0.02	− 0.79
H2	− 1.03	2.36	− 2.95	− 1.36	− 3.31	− 1.86	− 2.37	− 2.61	− 1.73	− 0.68	− 1.50
H3	− 1.40	2.42	− 4.25	− 2.87	− 4.23	− 4.76	− 6.67	− 3.27	− 3.64	− 1.93	− 2.98

To assess the impact of human activity, the *EF* was computed and presented in Table 2 and Fig. S7^59,60^. The range of Mn *EF* values was 0.29 to 1.32, Cu varied from 0.18 to 0.90, Zn varied from 0.63 to 2, Pb varied from 0 to 0.82, and Cd varied from 5.93 to 59.99. Similar to *I*_*geo*_ Cd, it has the highest *EF* of all the studied elements.

**Table 2:** The enrichment factor (*EF*) in sediment samples.

	As	Cd	Co	Cr	Cu	Mn	Ni	Pb	Zn	Fe	Al
A2	7.53	19.91	0.19	0.64	0.55	0.45	0.39	0.00	1.08	1.32	1.00
A3	7.59	59.99	0.61	0.62	0.90	0.41	0.65	0.00	2.00	1.54	1.00
B1	1.14	10.28	0.45	1.03	0.22	0.80	0.72	0.49	0.94	1.60	1.00
B2	1.14	8.35	0.73	0.84	0.22	0.68	0.70	0.37	0.94	1.59	1.00
B3	0.88	6.69	0.45	0.69	0.30	0.71	0.76	0.34	1.08	1.29	1.00
C1	1.14	4.22	0.31	0.62	0.30	0.52	0.69	0.35	0.96	1.87	1.00
C2	0.88	6.82	0.41	0.91	0.27	0.67	0.75	0.40	1.02	1.41	1.00
C3	0.77	6.31	0.29	0.65	0.30	0.77	0.78	0.28	0.98	1.35	1.00
D1	0.76	7.85	0.38	0.68	0.29	1.00	0.67	0.27	0.92	1.31	1.00
D2	0.69	5.93	0.50	0.64	0.21	0.68	0.56	0.26	0.82	1.56	1.00
D3	7.85	48.76	0.16	0.51	0.40	1.22	0.63	0.26	1.17	2.44	1.00
E1	1.35	13.37	0.20	2.89	0.19	1.32	0.43	0.53	0.73	2.48	1.00
E2	1.40	11.47	0.48	0.93	0.25	0.70	0.69	0.53	1.03	1.50	1.00
E3	7.33	17.64	0.02	2.49	0.29	0.53	0.28	0.61	0.67	2.88	1.00
F1	1.40	8.14	0.63	1.48	0.24	0.92	0.73	0.46	1.02	1.48	1.00
F2	1.40	7.47	0.40	0.99	0.24	0.69	0.66	0.44	0.94	1.52	1.00
F3	1.41	6.83	0.17	0.51	0.23	0.48	0.58	0.43	0.87	1.56	1.00
G1	0.67	6.65	0.43	0.72	0.18	0.65	0.55	0.36	0.80	1.46	1.00
G2	1.16	12.55	0.16	0.69	0.26	0.64	0.69	0.41	0.99	1.67	1.00
G3	1.29	8.66	0.33	0.77	0.27	0.65	0.71	0.56	1.10	1.23	1.00
H1	1.04	8.54	0.36	1.11	0.26	0.89	0.65	0.39	0.90	1.70	1.00
H2	1.39	14.55	0.37	1.10	0.29	0.78	0.55	0.46	0.85	1.77	1.00
H3	3.00	42.12	0.42	1.08	0.42	0.29	0.08	0.82	0.63	2.07	1.00

Table S7 and Fig. S8 show no coastal sediment had been affected, with PLI levels ranging from 0.18 to 1.09^61^. Except for E2 and G3, all the sediments under study are clean. The range of *C*_*d*_ levels (Table S7 and Fig. S5) was 9.47 to 23.10 73. A comprehensive assessment of pollution that considers many heavy metals is provided by *C*_*d*_. *I*_*geo*_ offers a straightforward assessment, *EF* assesses regulatory compliance directly, and PLI offers a comprehensive analysis that considers various metal toxicities. The legal framework and specific evaluation objectives will dictate which of these indicators is used.

### Eco-toxicological sense of HM contamination

The ecotoxicological significance of HM contamination in sediments was evaluated based on sediment quality criteria designed for marine and estuarine habitats^68,69^. According to Table S8, these effects are the effect range low (*ERL*)/effect range median (*ERM*) and the threshold effect level (*TEL*)/probable effect level (*PEL*). *ERL* and* TEL* indicate chemical concentrations below the harmful effects on sediment-dwelling species, whereas* ERM* and* PEL* represent chemical concentrations that exceed the anticipated adverse effects. Sediment pollution can be evaluated by comparing various indications of contamination with effect-based sediment quality guidelines (*SQGs*). Numerical *SQGs* have been used to identify contaminants of concern in aquatic environments^69^ (Table S8). Sediments were categorized as non-contaminated, moderately polluted, or very polluted according to USEPA *SQGs*^70^ (Table S8). The investigated silt exhibited moderate contamination of Ni but was non-polluted with Cu and Zn by *SQG* norms. Additionally, compared to *TEL* and *ERL*, the *C*_*d*_ concentrations are greater (Table S8).

### Statistical analysis

#### Correlation coefficient analysis of the studied element and grain size analysis

Pearson’s correlation matrix assessed the identified metals’ correlation structure. High relationships between Co and Cu (0.666), Ga (0.763), Mn (0.729), V (0.878), W (0.945), Zn (0.769), and Al_2_O_3_ (0.779) are revealed by the correlation matrix analysis (Table S9). Strong correlations (*R*^2^ = 0.810, 0.945, 0.952, 0.957, and 0.912, respectively) are found between Cu and Mn, Nb, Ni, Zn, and Al. A robust correlation (*R*^2^ = 0.957) between Cu and Zn supports the previously reported observations that they exhibit comparable geochemical behaviour and co-occur in rock-forming minerals^71,72^. The descriptive statistics of the studied trace and major elements are present in Tables S10 and S11.

#### Principal component analysis (PCA)

Principal Component Analysis (PCA) serves as a versatile tool for modelling, classification, pattern recognition, and a range of data analysis tasks (Table S12 and Fig. S9)^73,74^. It is one of the most straightforward and traditional approaches to standard factor analysis. These elements, usually referred to as axes, may be useful for comparing or studying the characteristics of heavy metals as they represent orthogonal linear combinations of the observed variables. When the first two axes of the ordination function are displayed, data from experimental systems with similar attributes tend to group closely together, while data from systems with dissimilar attributes are located further apart. For trace elements, the first group (PC1) accounted for 43.831% of the total variance with a high loading of Ce, Cu, Ga, Mn, Nb, Ni, Pb, V, W, Zn, Fe, and Al, which showed a Strong positive loading (> 0.7) for Fe (0.916), Zn (0.969) and Al (0.982), Moderate positive loadings (0.5–0.7) for Sn (0.511) and Ti (0.563). This pattern suggests potential covariance between Fe/Cr and Ni/Zn, consistent with mixed lithogenic and anthropogenic influences across sampling sites. No universal correlation existed for all metals at all stages (Table S12). These metals have common source parts. The second component (PC2) accounted for 14.795% of the total variance with a high loading of Br, I, and Sc (0.851, 0.801, and 0.901), respectively. The third component (PC3) accounted for 8.023%, correlated with Ta (0.604). On the other hand, for major elements, the first group (PC1) accounted for 40.395% of the total variance with a high loading of SiO_2_, TiO_2_, Al_2_O_3_, Fe_2_O_3_, MgO, and SrO, which means that these metals were correlated at all stages (Table S13). The second component (PC2) accounted for 18.3% of the total variance with a high loading of MnO, ZnO, and ZrO (0.5901, − 0.703, and 0.776), respectively. The third component (PC3) accounted for 11.350% and was correlated with SO_3_ (0.905).

The Nile Delta coastal area is a vital socio-economic hub for Egypt. It is packed with diverse-sized urbanized areas and intertwining economic activities and resources, including agricultural land, industrial centers, aquaculture, coastal marine living and non-living resources, tourism, ports, and other activities. Subsequently, the Nile Delta coastal area is subjected to multiple environmental pressures, such as pollution, coastal degradation, erosion, and reduced productivity^12,75–77^. Run-offs from various agricultural, industrial, and municipal sources from the northern sector of the Nile Delta are collected through a complicated drainage network (Fig. 1). The drainage network, carrying partially treated wastes^78^, discharges into the Nile promontories and the coastal lagoons. These wastes are finally discharged into the Mediterranean via the coastal lagoon outlets or directly via drains (e.g. El-Tabia and Kitchener drains).

The evident influence of land-based activities on the Nile Delta coastal area has prompted the attention of several workers on the contamination of coastal sediments with trace metals^76–81^ and coastal lagoons^82–84^. These workers reported that the water and sediments of the Nile Delta coastal area, coastal lagoons, and some drains are polluted with trace metals. Abu Qir Bay, west of the Nile Delta and the Delta coastal lagoons, has been identified as a hotspot for metal pollution^85^. Pollution in this area corresponds to the intense anthropogenic land-based activities, including industry (such as foods, agricultural chemicals, textiles, leather tanning, and paper mills), agriculture, aquaculture, and municipal run-offs.

The findings align with previous studies identifying the Nile Delta coastal region as a hotspot for trace metal pollution^12,76–81^, except Zn, Cd, and Pb. Notably, Cd exhibits a higher average concentration, reaching 2.44 µg/g (Table S14). While the trace metal concentrations in Nile Delta coastal sediments are consistent with reported levels from other studies in the same region (Table S14), they are lower than those in adjacent coastal lagoons. These lagoons serve as the primary recipients of pollution from land-based activities in the Nile Delta, acting as reservoirs that retain contaminants before their release into the coastal zone. The results are comparable to those reported for other coastal regions with similar environmental conditions, such as the Simenit Lake and Yangtze River Delta, all receiving mixed runoff from industrial, agricultural, and urban sources.

### Proposed mitigation strategies

A combination of source control, remediation techniques, and policy interventions is essential to address heavy metal pollution in the Nile Delta. First, regulating industrial discharges and promoting cleaner agricultural practices are critical steps. Stricter enforcement of environmental standards can minimize the release of heavy metals such as Cr, Ni, Cu, and Zn from industries while encouraging the use of organic fertilizers and biopesticides can reduce inputs of Cd, Pb, and As from agricultural runoff. Improved urban waste management systems are also necessary to prevent the disposal of electronic waste and other hazardous materials into water bodies.

Second, sediment remediation techniques such as dredging, in-situ stabilization, and phytoremediation can help mitigate existing contamination. Removing heavily contaminated sediments from hotspots can prevent the re-suspension of toxic metals like As, Cd, and Pb, while amendments like lime or biochar can immobilize metals in sediments, reducing their bioavailability. Phytoremediation using metal-accumulating plants, such as water hyacinth, can further absorb and sequester heavy metals from contaminated areas. Additionally, ecosystem restoration through wetland construction and reforestation can act as natural filters, trapping pollutants and reducing erosion.

Finally, public awareness, enhanced monitoring, and robust policy frameworks are vital for long-term solutions. Educational campaigns can inform local communities about the risks of heavy metal pollution, while stakeholder collaboration can foster collective action. Establishing comprehensive monitoring networks and geospatial tools can help identify pollution hotspots and prioritize mitigation efforts. Strengthening environmental regulations and incentivizing sustainable practices will ensure that industries and farmers adopt cleaner technologies, ultimately protecting the Nile Delta ecosystem and promoting sustainable development.

## Conclusions

The samples collected were analyzed for both Trace elements, which are Transition metals (Ag, Cu, V, Cr, Mo, Co, Ni, Hf, Mn, W, Sc, Y, Fe, Zr, Nb, Ta), Post transition metals (Tl, Bi, Cd, Ga, Pb, Sn, Zn, Al), Alkali/Alkaline earth (Rb, Cs, Sr, Ba), Metalloids (As, Ge, Sb, Te), Reactive nonmetals (Br, I, Se), Rare earth element (Actinides U and Th), Rare earth element Lanthanides (Ce, La, Nd, Sm), and Major elements which are SiO_2_, TiO_2_, Al_2_O_3_, Fe_2_O_3_, MnO, CaO, MgO, P_2_O_5_ etc. and minerals in water stream sediments collected from Nile delta. Transition metals analysis results obtained from eight sections differed from each other. For example, while the maximum concentration of V metal (116.9 mg kg^–1^) was observed in the G2 and G3 sections, its minimum concentration (31 mg kg^–1^) was observed in the A3, E3, and H3 sections. Cobalt metal was observed with its maximum concentration (13.2 mg kg^–1^) in the B2 section, while its minimum concentration (0.8 mg kg^–1^) was observed in the A2 section. All transition metals were detected in concentrations ranging from 0.1 to 569.3 mg kg^–1^ in different sections.

Furthermore, the proportion of clay and sand in the analyzed sections exhibited significant variations in the results obtained from wet Particle Size Analysis (PSA). The sand percentage varies from 3.56% in the G3 section to 95.49% in the E1 section, while the clay percentage varies from 0.27% in the E3 section to 12.50% in the D3 section. In the sediments, Quartz (6.7–91.4%), Aragonite (4.9–77%), and Calcite (5.2–33%) minerals, as well as Albite (21.7–48.1%), microcline (Feldspar minerals such as (12.1–30.2%) and illite (0–26.4%) were also detected. According to this study, the mean *I*_geo_ of these elements is less than zero (*I*geo < 0), which suggests that heavy metal contamination of the Nile Delta appears to have been minimal overall. Cadmium is moderately to heavily polluted in all studied locations. The *PLI* results, which showed no coastal sediment had been affected, varied from 0.18 to 1.09.

While these indices provide useful baseline insights, we acknowledge the limitations of relying solely on them without supporting biological or ecological risk data. As such, the conclusions have been cautiously framed to reflect a preliminary contamination assessment, consistent with current scientific practices in the absence of region-specific geochemical baselines or source apportionment analysis.

## Electronic supplementary material

Below is the link to the electronic supplementary material.

Supplementary Material 1

## Data Availability

Data will be made available upon request from the corresponding author.
