# Supplementary material for: Distribution and ecotoxicological assessment of trace elements and minerals in Nile Delta sediments using multitechnique analysis
Source: Sci Rep. 2025 Jul 17;15:25933. doi: 10.1038/s41598-025-09940-w (PMC12271327; doi:10.1038/s41598-025-09940-w)
Supplement: Supplementary file 1 — Supplementary Material 1 [file 41598_2025_9940_MOESM1_ESM.docx]

**Supplementary material**

Distribution and Ecotoxicological Assessment of Trace Elements and Minerals in Nile Delta Sediments Using Multitechnique Analysis

Mohamed A. Hassaan^a^, Marwa R. ElKatory^b^, Wael Abdelwahab^c^, Murat Yılmaz^d^, Ahmed El Nemr^a,^*

^a^Environment Division, National Institute of Oceanography and Fisheries (NIOF), Kayet Bey, Elanfoushy, P.O. 21556, Alexandria, Egypt.

^b^Advanced Technology and New Materials Research Institute, SRTA-City, New Borg El-Arab City 21934, Alexandria, Egypt

^c^Geological Sciences Department, National Research Centre, El-Buhouth St., Dokki, Cairo 12622, Egypt

^d^Osmaniye Korkut Ata University, Bahçe Vocational School, Department of Chemistry and Chemical Processing Technologies, Osmaniye, 80000, Türkiye

Authors email: [mhss95@mail.com](mailto:mhss95@mail.com) (M.A. Hassaan); [marwa_elkatory@yahoo.com](mailto:marwa_elkatory@yahoo.com) (M.R. El-Katory); [dr.wael.nrc@gmail.com](mailto:dr.wael.nrc@gmail.com) (W. Abdelwahab); [muratyilmaz@osmaniye.edu.tr](mailto:muratyilmaz@osmaniye.edu.tr) (M. Yılmaz)

*Corresponding author: [ahmedmoustafaelnemr@yahoo.com](mailto:ahmedmoustafaelnemr@yahoo.com); [ahmed.m.elnemr@gmail.com](mailto:ahmed.m.elnemr@gmail.com)

**
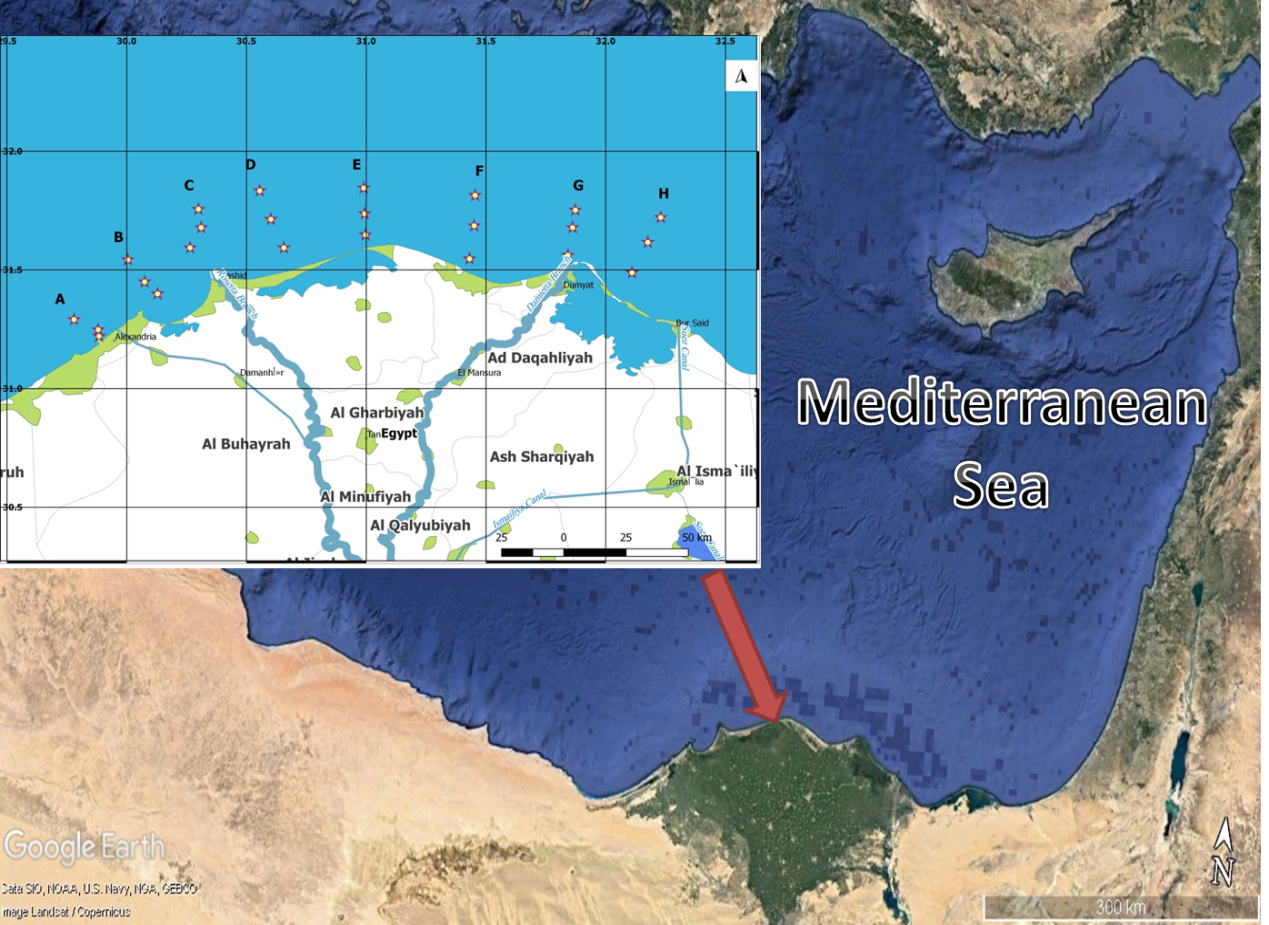
**

Fig. S1. The map of the study area of Nile Delta, Mediterranean Sea, Egypt. Software QGIS 3.18; <https://www.filehorse.com/download-qgis/61739/>)

| 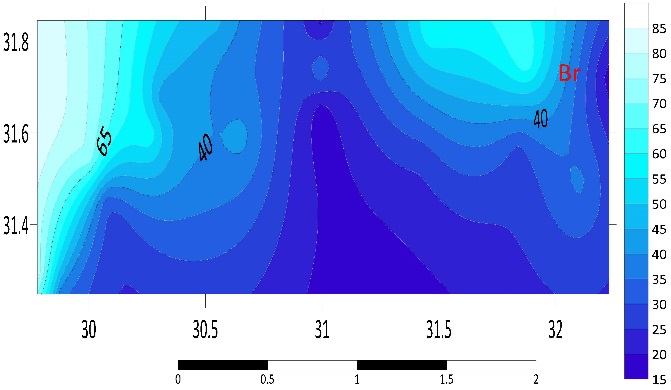 | 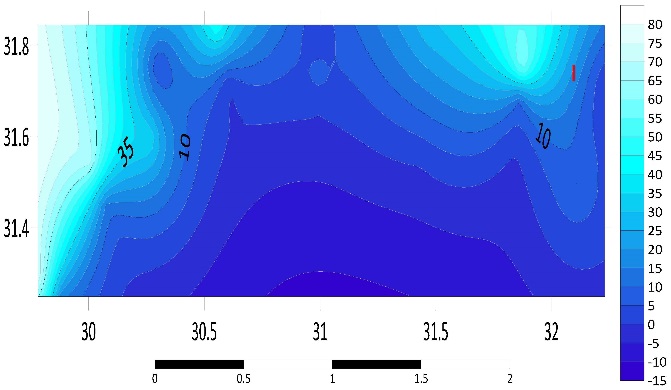 |
| --- | --- |
| 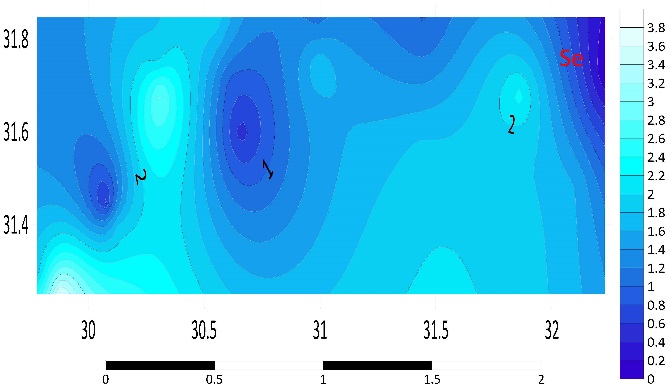 |  |

Fig. S2. Distribution Map of reactive nonmetals (Br, I, Se) in the investigated area.

| **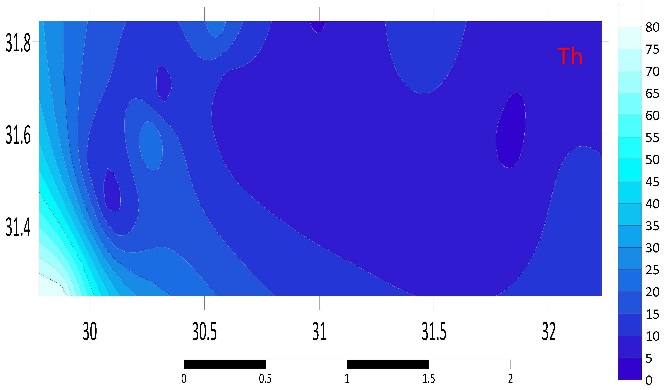** | **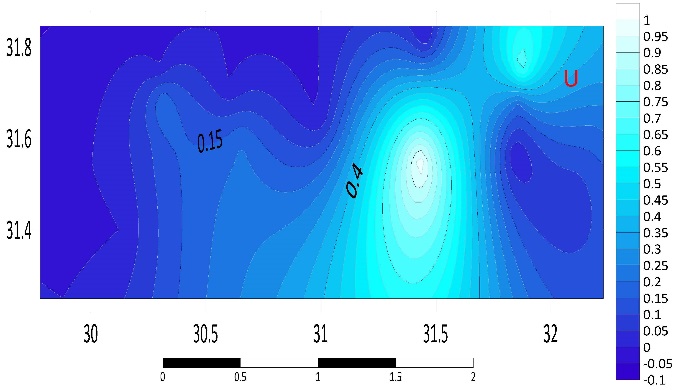** |
| --- | --- |

Fig. S3. Distribution Map of rare earth elements (Th and U) in the investigated area.

| 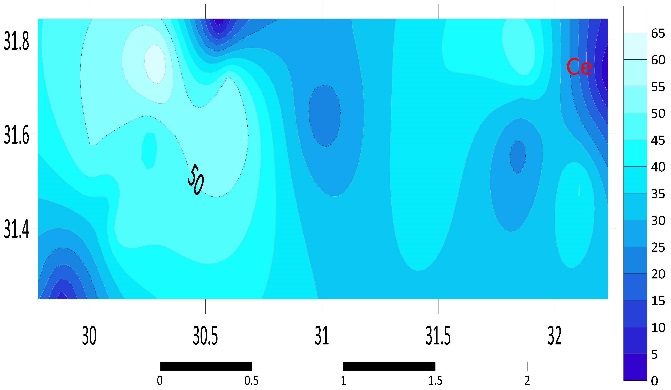 | 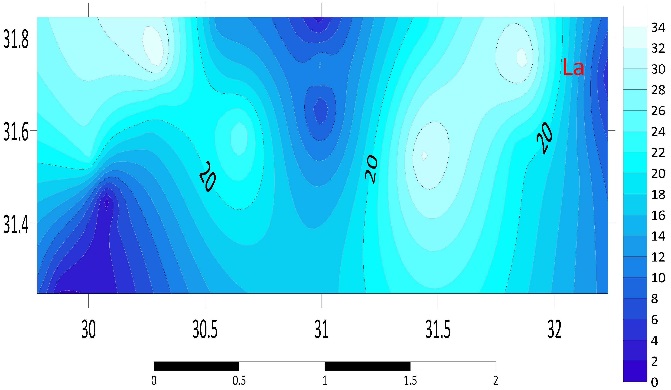 |
| --- | --- |
| 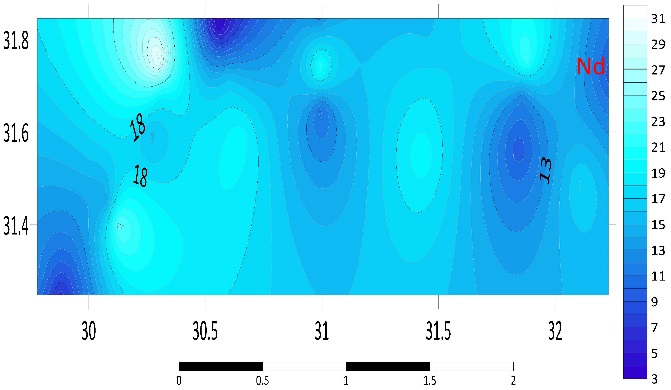 | 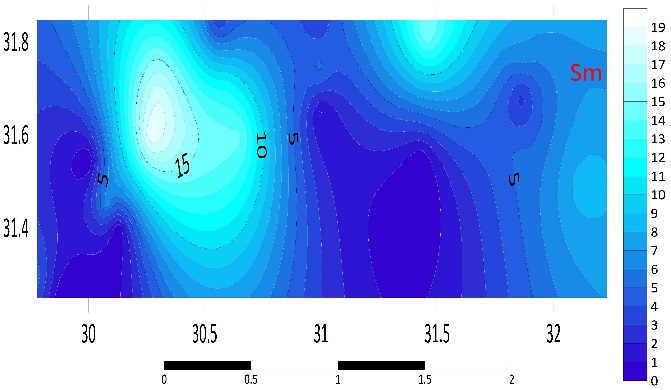 |

Fig. S4. Distribution Map of rare earth element Lanthanides (Ce, La, Nd, Sm) in the investigated area.

| 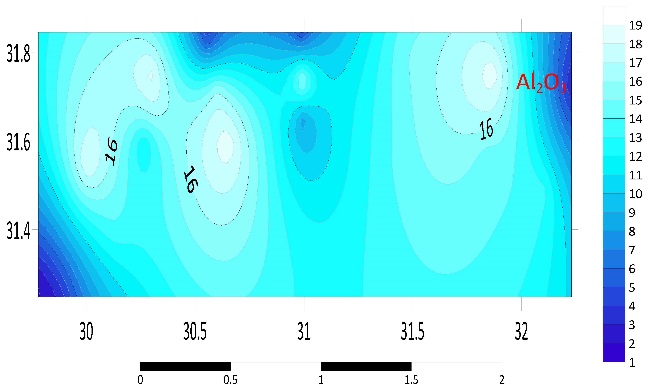 | 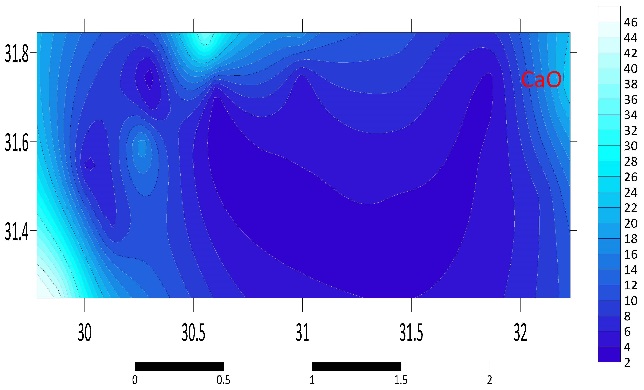 |
| --- | --- |
| 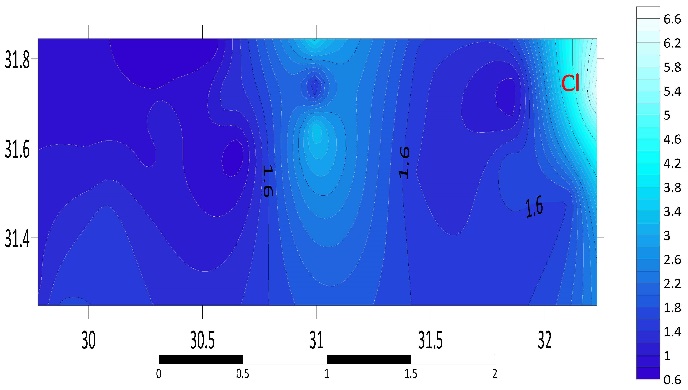 | 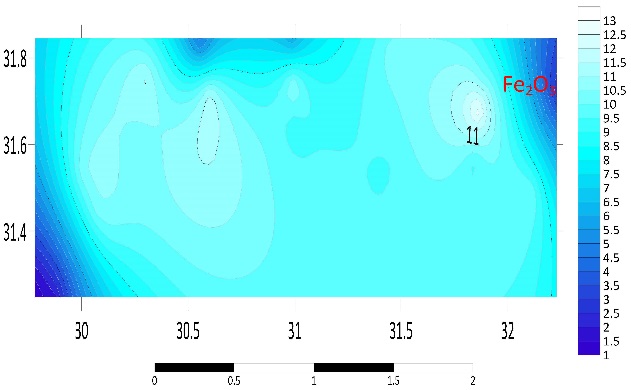 |
| 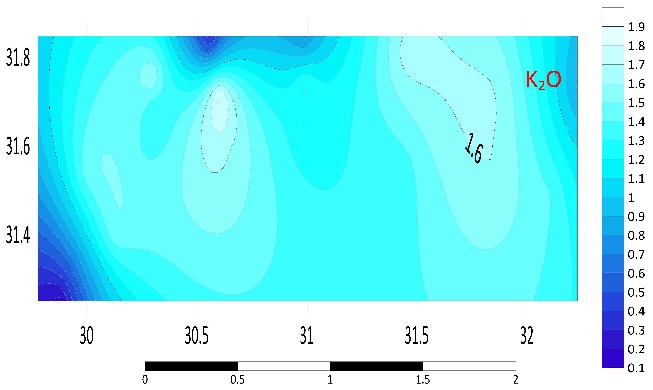 | 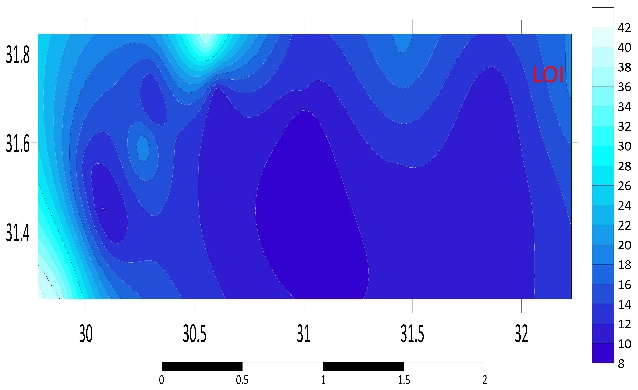 |
| 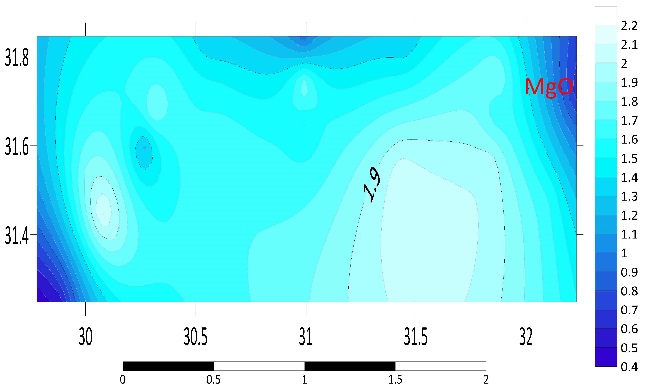 | 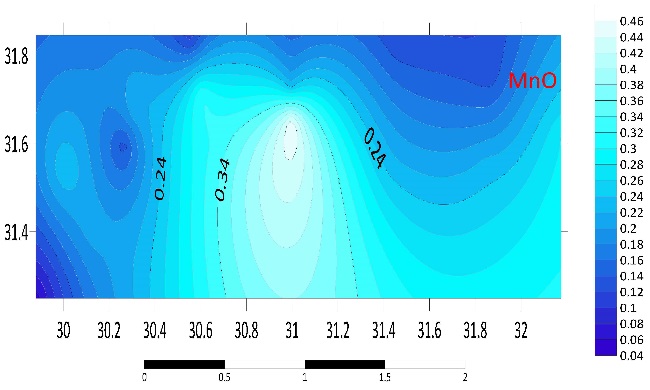 |
| 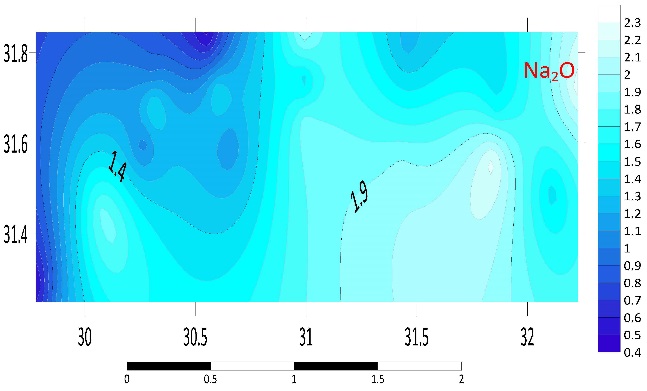 | 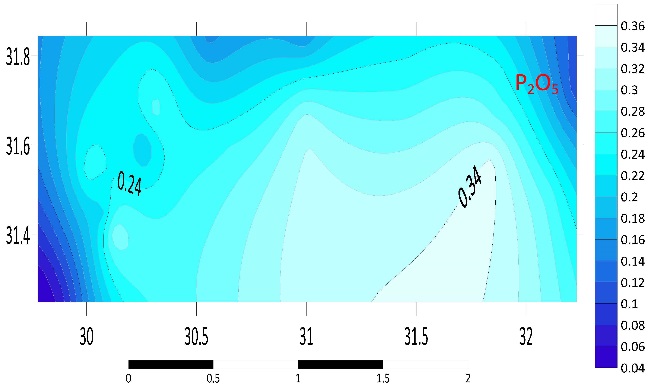 |
| 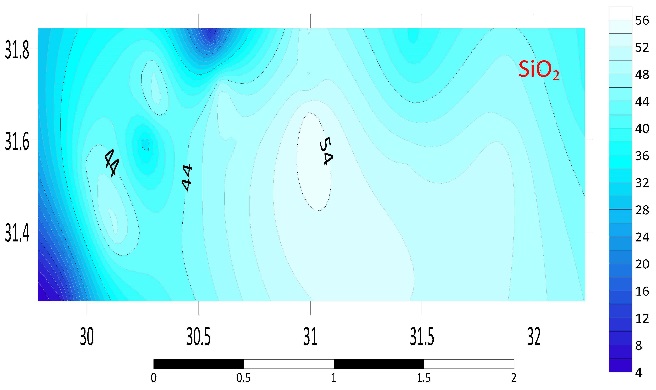 | 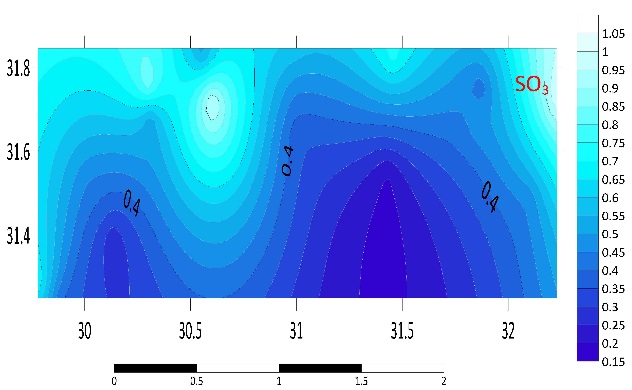 |
| 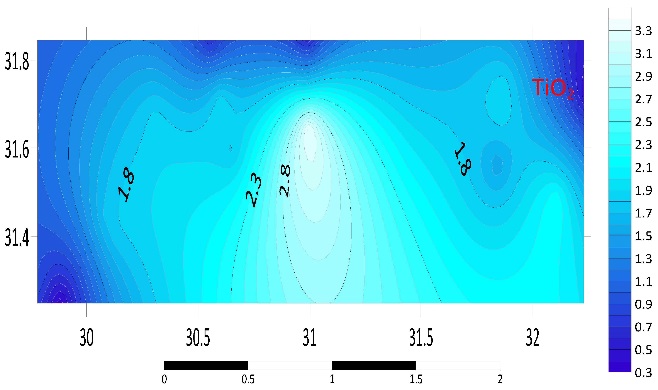 |  |

Fig. S5. Distribution Map of major elements (Al_2_O_3_, CaO, Cl, Fe_2_O_3_, K_2_O, MgO, MnO, Na_2_O, P_2_O_5_, SiO_2_, SO_3_, TiO_2_) in the investigated area.

| 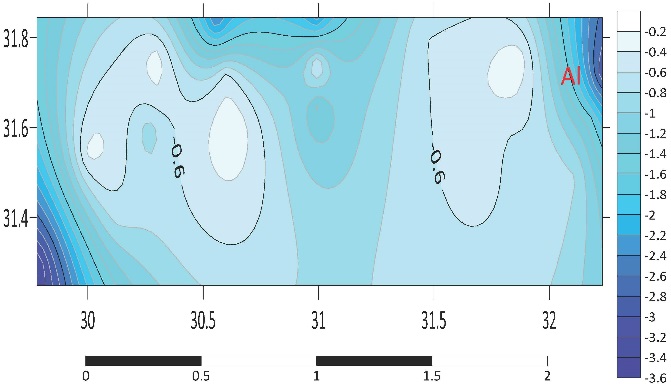 | 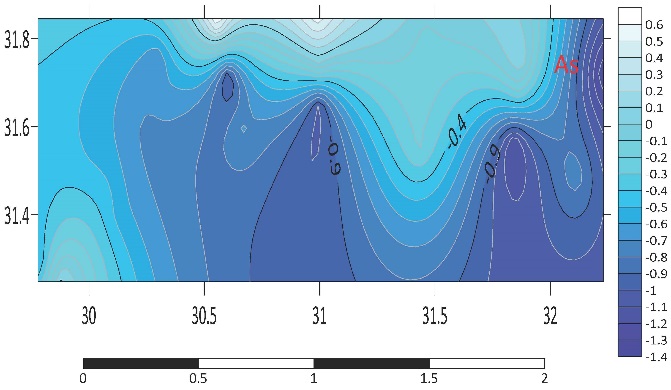 |
| --- | --- |
| 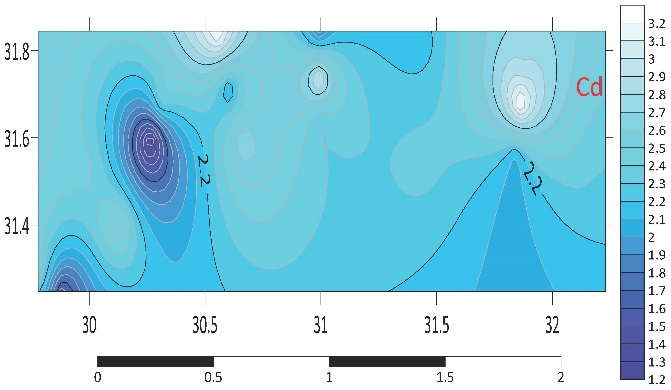 | 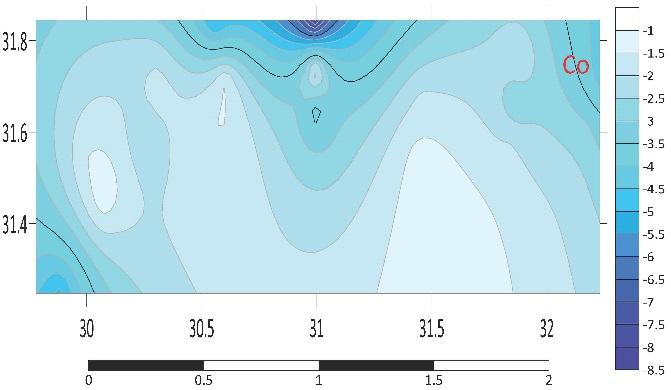 |
| 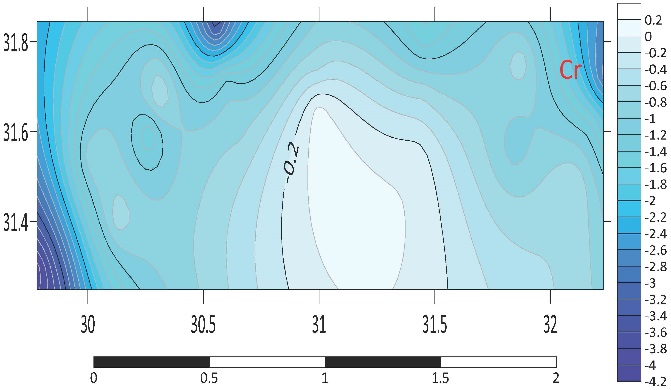 | 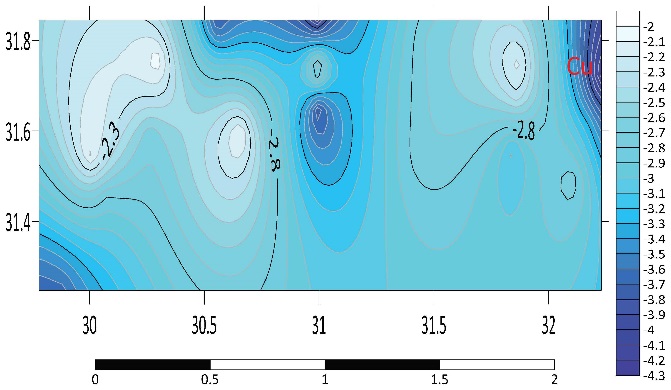 |
| 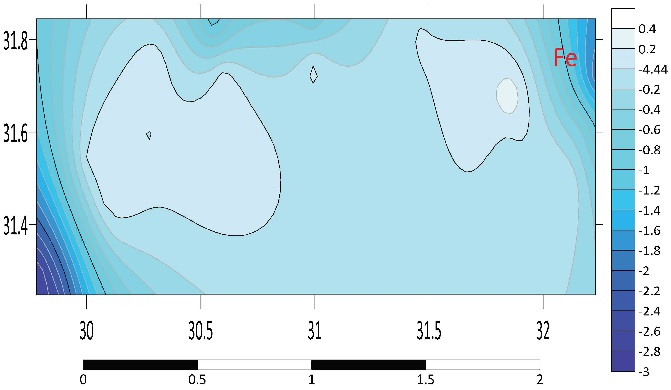 | 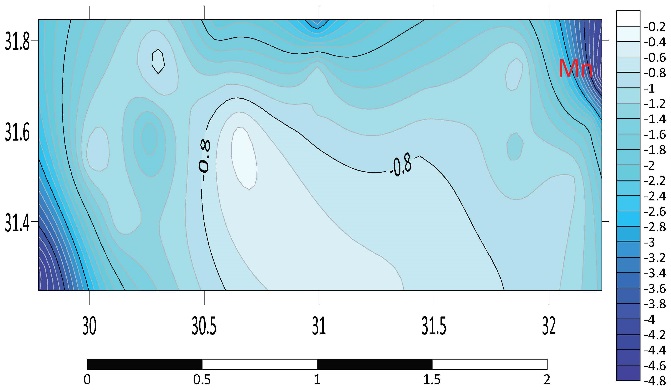 |
| 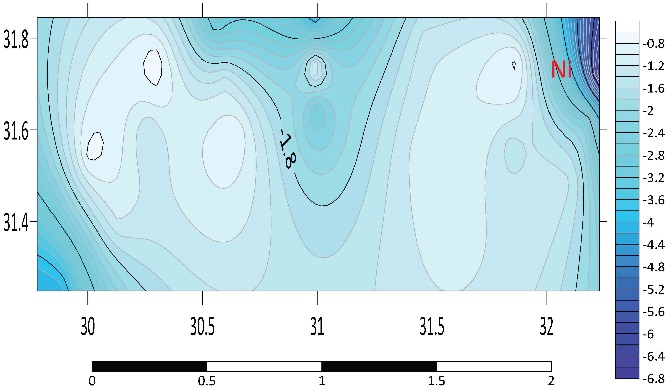 | 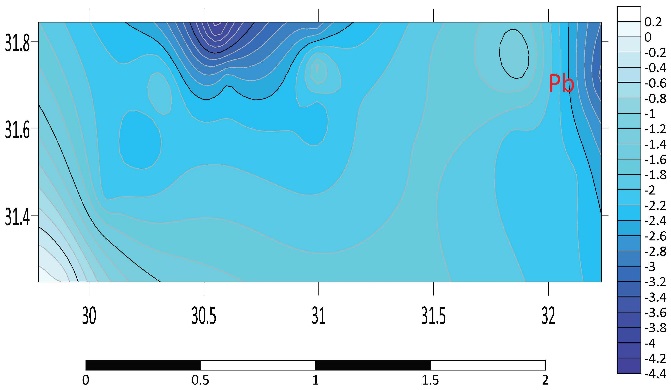 |
| 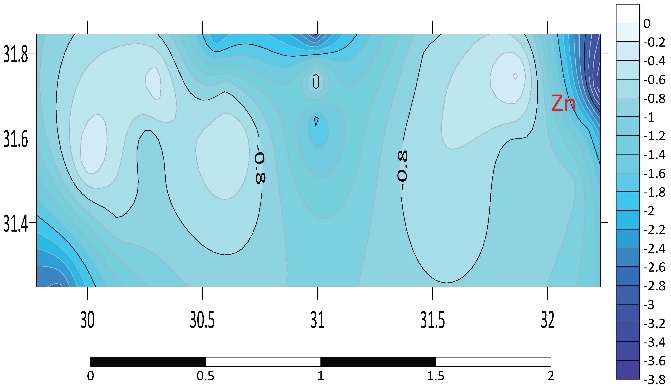 |  |

Fig. S6. Distribution map of the calculated *I*_geo_ values for the sediments in the investigated area.

| 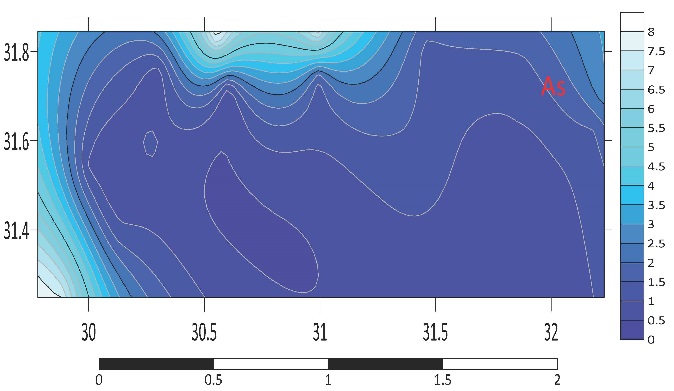 | 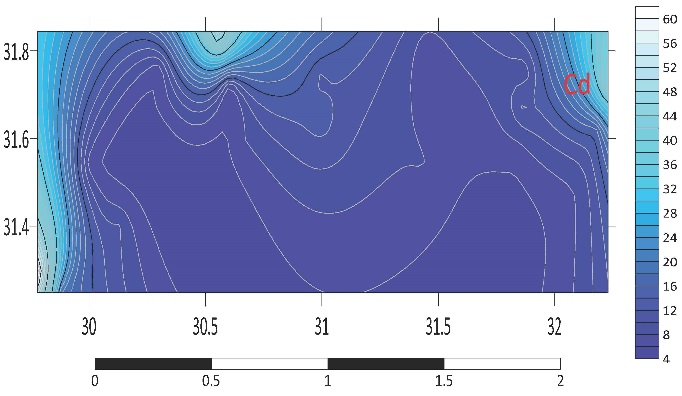 |
| --- | --- |
| 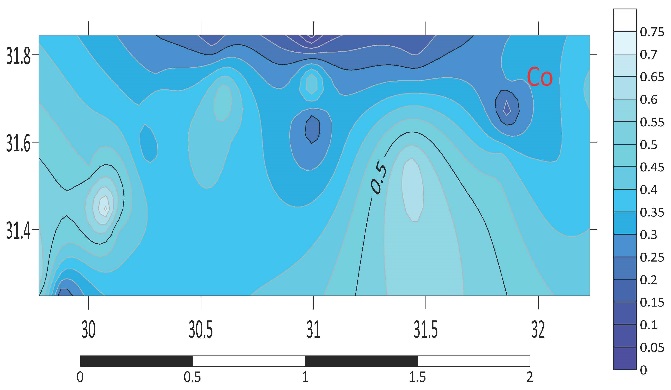 | 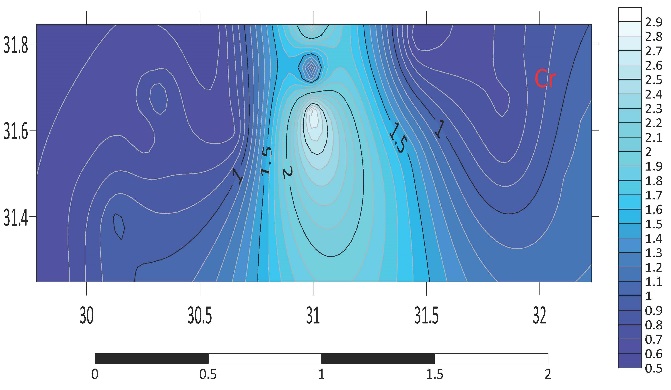 |
| 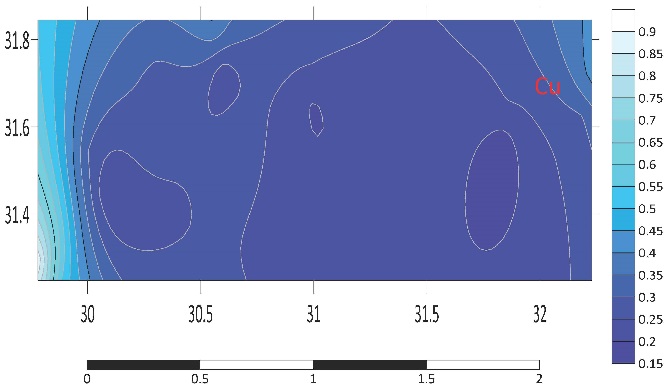 | 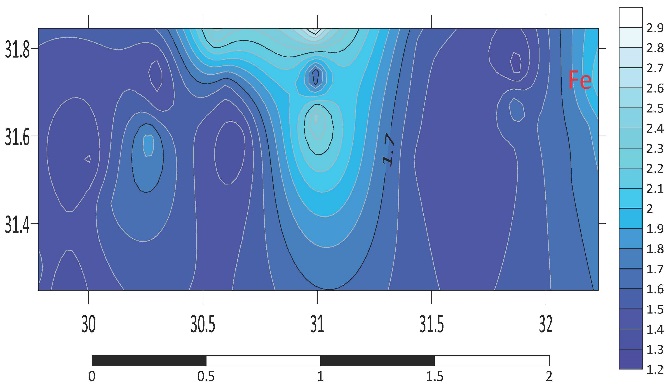 |
| 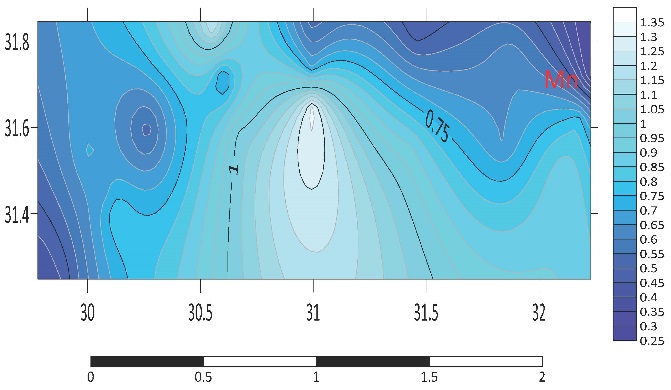 | 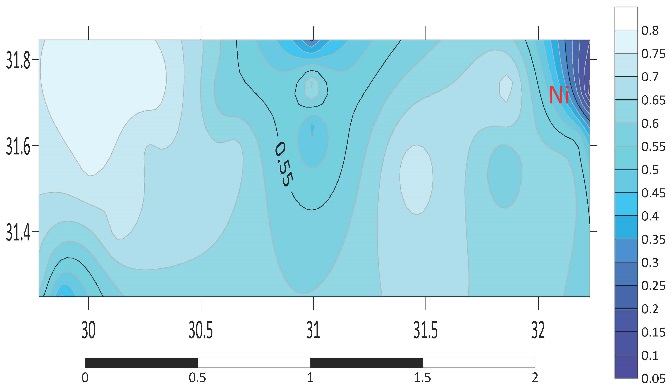 |
| 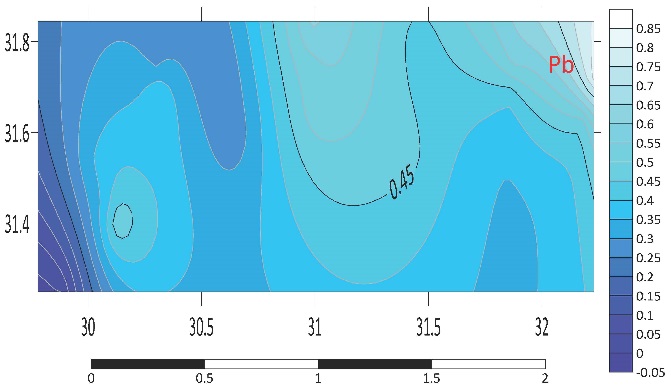 | 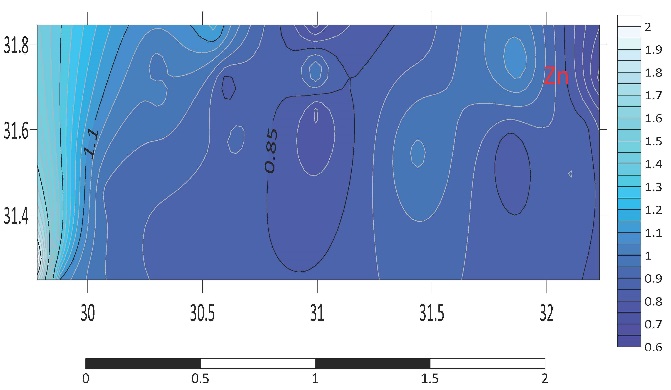 |

Fig. S7. Distribution Map of the calculated EF values for the sediments in the investigated area.

| 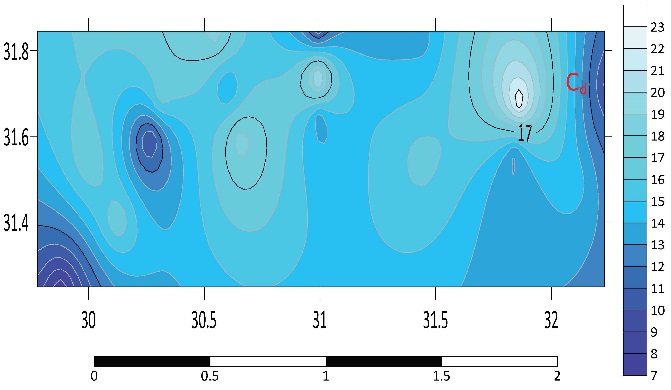 | 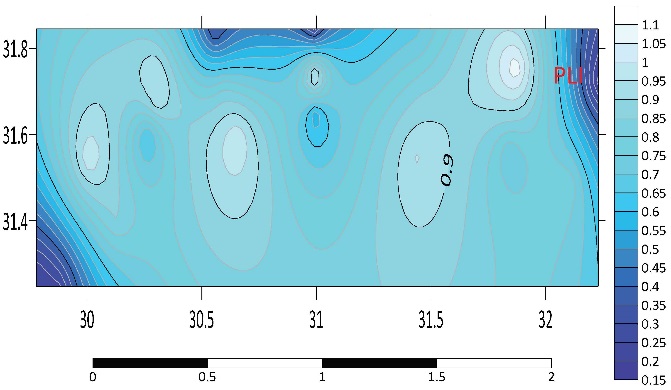 |
| --- | --- |

Fig. S8. Distribution Map of the calculated *C*_d_ and *PLI* values for the sediments in the investigated area.

| 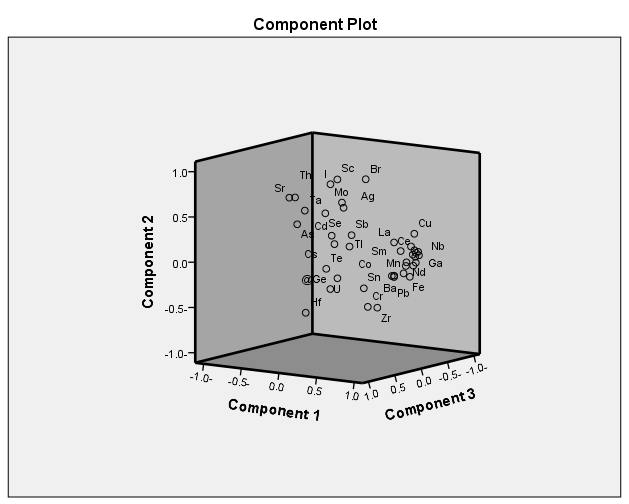 | 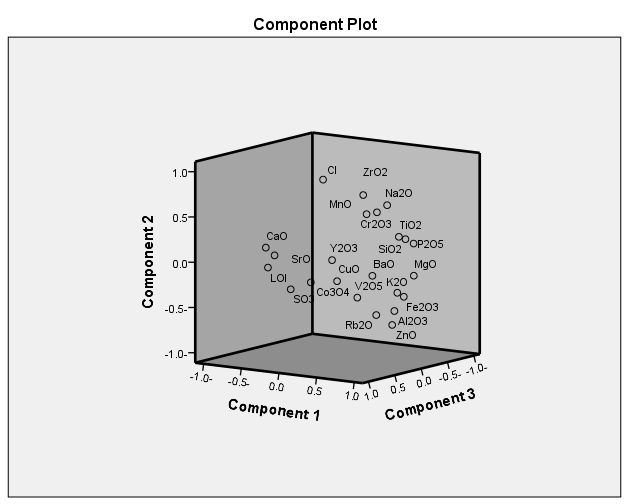 |
| --- | --- |
| **a** | **b** |

Fig. S9. Principle component analysis (a) trace elements, and (b) major elements.

Table S1. The sample stations are described below (Depth and geographic location).

| Location | Long | Lat | Depth | Area | | |
| --- | --- | --- | --- | --- | --- | --- |
| A1 | 29.8846052 | 31.22092062 | 10 | WH |  |  |
| A2 | 29.88072909 | 31.24805334 | 30 |  |  |  |
| A3 | 29.77995043 | 31.29069046 | 50 |  |  |  |
| B1 | 30.12879966 | 31.39922133 | 10 | Abu Qir |  |  |
| B2 | 30.07453422 | 31.44961067 | 30 |  |  |  |
| B3 | 30.00476438 | 31.54263713 | 50 |  |  |  |
| C1 | 30.26446325 | 31.59302646 | 10 | Rasheed Nile branch | |  |
| C2 | 30.31097648 | 31.67830072 | 30 |  |  |  |
| C3 | 30.29934817 | 31.75582277 | 50 |  |  |  |
| D1 | 30.6559496 | 31.59302646 | 10 | Burullus |  |  |
| D2 | 30.60168417 | 31.71318564 | 30 |  |  |  |
| D3 | 30.55517094 | 31.83334482 | 50 |  |  |  |
| E1 | 30.99704663 | 31.6472919 | 10 | Balteem |  |  |
| E2 | 30.99317052 | 31.73644225 | 30 |  |  |  |
| E3 | 30.98929442 | 31.84497313 | 50 |  |  |  |
| F1 | 31.43117011 | 31.54651323 | 10 | Mansourah elgedida and Gamasa | | |
| F2 | 31.45055062 | 31.68605292 | 30 |  |  |  |
| F3 | 31.45442672 | 31.81396431 | 50 |  |  |  |
| G1 | 31.84203698 | 31.56201764 | 10 | Dameitta Nile branch Ras elbar | | |
| G2 | 31.86141749 | 31.67830072 | 30 |  |  |  |
| G3 | 31.8730458 | 31.75194666 | 50 |  |  |  |
| H1 | 32.10948805 | 31.48837169 | 10 | Manzala Lake | |  |
| H2 | 32.1753818 | 31.61628308 | 30 |  |  |  |
| H3 | 32.22964723 | 31.72093784 | 50 |  |  |  |

Table S2. The trace metals concentration (mg kg^-1^) in marine sediments.

|  | Ag | As | Ba | Bi | Br | Cd | Ce | Co | Cr | Cs | Cu | Ga | Ge | Hf |
| --- | --- | --- | --- | --- | --- | --- | --- | --- | --- | --- | --- | --- | --- | --- |
| **A2** | 1.10 | 21.30 | ND | 1.80 | 36.60 | 1.30 | 6.40 | 0.80 | 12.50 | 5.10 | 5.40 | 3.40 | ND | 7.60 |
| **A3** | 1.20 | 13.70 | ND | ND | 79.70 | 2.50 | 26.20 | 1.60 | 7.70 | 7.50 | 5.60 | 3.80 | ND | 4.00 |
| **B1** | 1.30 | 13.40 | 126.10 | 1.60 | 27.50 | 2.80 | 49.10 | 7.80 | 83.80 | 11.00 | 8.80 | 16.80 | ND | 4.90 |
| **B2** | 1.10 | 14.20 | 136.40 | 1.90 | 31.60 | 2.40 | 37.90 | 13.20 | 72.40 | 4.60 | 9.50 | 18.20 | ND | 9.40 |
| **B3** | 1.30 | 13.60 | 96.40 | 1.40 | 72.70 | 2.40 | 49.70 | 10.20 | 73.80 | 12.20 | 15.90 | 24.90 | ND | 7.50 |
| **C1** | 0.60 | 11.70 | 28.00 | 0.10 | 58.40 | 1.00 | 42.80 | 4.70 | 44.20 | 10.20 | 10.50 | 16.30 | ND | 1.20 |
| **C2** | 0.90 | 12.80 | 103.50 | 1.00 | 42.20 | 2.30 | 57.10 | 8.70 | 91.60 | 4.00 | 13.70 | 21.80 | ND | 5.80 |
| **C3** | 1.50 | 12.10 | 83.90 | 2.80 | 50.30 | 2.30 | 65.40 | 6.80 | 71.10 | ND | 16.50 | 27.60 | ND | 5.70 |
| **D1** | 1.10 | 12.20 | 69.30 | 0.60 | 42.30 | 2.90 | 52.60 | 8.90 | 75.30 | 8.80 | 16.10 | 23.80 | ND | 4.10 |
| **D2** | 0.70 | 9.60 | 74.40 | 1.10 | 35.60 | 1.90 | 53.70 | 10.20 | 61.60 | 4.20 | 9.90 | 18.10 | 0.20 | 6.60 |
| **D3** | 1.50 | 29.30 | ND | 1.30 | 45.60 | 4.20 | ND | 0.90 | 13.10 | 4.80 | 5.20 | 4.10 | ND | 4.50 |
| **E1** | 1.10 | 9.60 | ND | 0.80 | 16.70 | 2.20 | 19.70 | 2.10 | 142.70 | 4.00 | 4.80 | 7.70 | ND | 14.50 |
| **E2** | 1.20 | 19.00 | 63.30 | 2.80 | 34.30 | 3.60 | 28.00 | 9.60 | 87.90 | 5.20 | 11.60 | 20.80 | ND | 8.50 |
| **E3** | 0.70 | 28.80 | 35.60 | ND | 18.50 | 1.60 | 29.00 | 0.10 | 67.80 | 9.80 | 4.00 | 3.50 | ND | 7.10 |
| **F1** | 0.60 | 17.10 | 74.70 | 2.70 | 26.80 | 2.30 | 39.80 | 11.30 | 125.50 | 4.60 | 10.10 | 18.90 | 0.60 | 9.60 |
| **F2** | 0.80 | 17.50 | 58.15 | 2.55 | 42.40 | 2.15 | 39.45 | 7.25 | 85.20 | 4.95 | 10.20 | 18.10 | 0.30 | 8.30 |
| **F3** | 1.00 | 17.90 | 41.60 | 2.40 | 58.00 | 2.00 | 39.10 | 3.20 | 44.90 | 5.30 | 10.30 | 17.30 | ND | 7.00 |
| **G1** | 0.80 | 8.30 | 131.80 | 3.30 | 28.10 | 1.90 | 22.20 | 7.70 | 62.00 | ND | 7.70 | 16.90 | ND | 5.90 |
| **G2** | 1.60 | 17.20 | 69.60 | 3.30 | 48.40 | 4.30 | 34.50 | 3.50 | 70.70 | 5.20 | 13.50 | 23.40 | ND | 6.60 |
| **G3** | 1.70 | 20.60 | 86.20 | 5.30 | 64.20 | 3.20 | 51.70 | 7.70 | 85.50 | ND | 15.10 | 26.50 | ND | 5.70 |
| **H1** | 1.03 | 11.73 | 55.85 | 0.78 | 36.23 | 2.23 | 41.05 | 5.88 | 86.50 | 8.50 | 10.05 | 16.15 | ND | 6.18 |
| **H2** | 0.91 | 9.56 | 36.58 | 0.78 | 26.71 | 2.31 | 20.53 | 3.69 | 52.50 | 6.95 | 6.83 | 9.83 | ND | 7.24 |
| **H3** | 0.80 | 7.40 | 17.30 | - | 17.20 | 2.40 | ND | 1.50 | 18.50 | 5.40 | 3.60 | 3.50 | ND | 8.30 |

Table S2. Continued.

|  | Hg | I | La | Mn | Mo | Nb | Nd | Ni | Pb | Rb | Sb | Sc | Se | Sm |
| --- | --- | --- | --- | --- | --- | --- | --- | --- | --- | --- | --- | --- | --- | --- |
| **A2** | ND | 16.40 | 1.60 | 83.00 | 0.30 | ND | 6.30 | 5.70 | ND | ND | 4.50 | 24.70 | 3.70 | ND |
| **A3** | ND | 75.90 | 10.50 | 48.70 | 1.10 | ND | 12.30 | 6.10 | ND | ND | 4.50 | 29.00 | 1.90 | 4.60 |
| **B1** | ND | 6.30 | 6.60 | 617.00 | 0.70 | 13.00 | 23.80 | 44.40 | 8.90 | 34.70 | ND | 20.10 | 1.80 | ND |
| **B2** | ND | 13.90 | 0.70 | 555.50 | 1.00 | 13.30 | 14.20 | 45.70 | 7.00 | 35.80 | 4.40 | 18.20 | 0.50 | 6.70 |
| **B3** | ND | 67.40 | 24.50 | 720.60 | 1.90 | 24.80 | 16.10 | 61.90 | 8.20 | 54.40 | ND | 23.80 | 1.10 | ND |
| **C1** | ND | 34.80 | 17.60 | 350.80 | 1.40 | 13.10 | 15.50 | 37.20 | 5.60 | 27.60 | 7.10 | 17.80 | 2.50 | 19.00 |
| **C2** | ND | 13.30 | 23.40 | 643.60 | 0.90 | 23.40 | 19.00 | 57.10 | 9.10 | 45.90 | ND | 19.10 | 2.90 | 17.90 |
| **C3** | ND | 4.40 | 34.30 | 798.80 | 0.90 | 28.00 | 31.40 | 64.40 | 6.90 | 64.50 | 5.50 | 22.20 | 1.90 | 15.30 |
| **D1** | ND | ND | 25.90 | 1043.00 | 0.60 | 24.70 | 19.60 | 56.20 | 6.70 | 53.90 | 3.80 | 19.10 | 0.50 | 14.30 |
| **D2** | ND | ND | 17.90 | 613.20 | ND | 19.60 | 16.30 | 40.50 | 5.60 | 40.20 | 3.20 | 12.40 | 1.00 | 11.20 |
| **D3** | ND | 44.50 | 12.80 | 297.20 | 1.80 | 2.00 | 3.00 | 12.30 | 1.50 | 3.00 | ND | 26.50 | 1.90 | 3.10 |
| **E1** | ND | ND | 5.90 | 616.50 | 0.90 | 12.50 | 10.40 | 15.90 | 5.80 | 10.20 | ND | 12.10 | 1.50 | 0.70 |
| **E2** | ND | 7.00 | 13.00 | 618.60 | 1.30 | 20.40 | 21.40 | 49.00 | 11.00 | 40.40 | 0.20 | 20.70 | 1.80 | 5.40 |
| **E3** | ND | ND | 2.60 | 135.90 | 0.80 | 1.80 | 12.60 | 5.80 | 3.70 | 6.10 | ND | 9.20 | 1.10 | 3.00 |
| **F1** | ND | 0.70 | 32.50 | 733.10 | 0.30 | 15.90 | 19.90 | 46.70 | 8.70 | 32.50 | 6.00 | 18.80 | 1.90 | ND |
| **F2** | ND | 13.25 | 27.35 | 563.65 | 0.70 | 16.20 | 17.85 | 42.75 | 8.50 | 36.05 | 3.55 | 17.10 | 1.45 | 7.60 |
| **F3** | ND | 25.80 | 22.20 | 394.20 | 1.10 | 16.50 | 15.80 | 38.80 | 8.30 | 39.60 | 1.10 | 15.40 | 1.00 | 15.20 |
| **G1** | ND | ND | 20.90 | 523.30 | ND | 11.70 | 9.20 | 35.50 | 6.80 | 35.10 | 1.90 | 13.30 | 1.80 | 5.20 |
| **G2** | ND | 9.50 | 28.30 | 625.40 | 1.40 | 22.50 | 12.20 | 53.80 | 9.40 | 45.70 | 11.60 | 19.60 | 2.30 | 2.40 |
| **G3** | ND | 59.90 | 33.10 | 677.10 | 1.10 | 25.90 | 22.20 | 59.80 | 13.80 | 57.70 | 1.40 | 21.50 | 1.80 | 6.20 |
| **H1** | ND | 10.28 | 14.00 | 656.83 | 0.90 | 15.83 | 17.33 | 38.43 | 6.75 | 31.60 | 2.73 | 17.28 | 1.58 | 8.50 |
| **H2** | ND | 5.14 | 9.25 | 352.01 | 0.70 | 7.96 | 13.36 | 19.71 | 4.93 | 18.15 | 1.41 | 11.04 | 0.79 | 7.65 |
| **H3** | ND | ND | 4.50 | 47.20 | 0.50 | 0.10 | 9.40 | 1.00 | 3.10 | 4.70 | 0.10 | 4.80 | ND | 6.80 |

Table S2. Continued.

|  | Sn | Sr | Ta | Te | Th | Tl | U | V | W | Y | Yb | Zn | Zr | Fe | Al |
| --- | --- | --- | --- | --- | --- | --- | --- | --- | --- | --- | --- | --- | --- | --- | --- |
| **A2** | 5.10 | 5592.70 | 4.90 | 7.50 | 79.10 | 9.40 | ND | 6.90 | 8.60 | 0.50 | 0 | 22.4 | 0 | 13512.75 | 17411.57 |
| **A3** | ND | 5566.70 | 3.90 | ND | 75.90 | 9.30 | ND | 4.40 | 2.50 | 0.90 | 0 | 26.4 | 32.3 | 10099.59 | 11113.77 |
| **B1** | 11.90 | 674.70 | ND | 2.40 | 11.40 | 10.30 | ND | 93.70 | 36.10 | 24.00 | 0 | 80.7 | 290.9 | 68612.87 | 72652.3 |
| **B2** | 8.80 | 746.50 | 2.60 | 12.90 | 8.50 | 12.70 | ND | 95.90 | 37.80 | 24.00 | 0 | 85.1 | 242 | 71767.24 | 76632.09 |
| **B3** | 10.70 | 684.60 | 3.30 | ND | 12.20 | 14.60 | ND | 115.60 | 51.10 | 31.60 | 0 | 122.4 | 269.2 | 72494.64 | 95610.18 |
| **C1** | 7.50 | 1879.10 | 1.00 | 5.70 | 25.90 | 8.40 | 0.10 | 60.30 | 26.80 | 18.70 | 0 | 72.1 | 155.3 | 69801.88 | 63221.48 |
| **C2** | 1.60 | 392.10 | 2.60 | 1.30 | 8.50 | 11.10 | 0.20 | 114.70 | 47.60 | 30.10 | 0 | 108.7 | 289.4 | 74718.78 | 89878.65 |
| **C3** | 8.00 | 225.20 | ND | 5.40 | 10.10 | 8.60 | ND | 108.00 | 48.90 | 36.80 | 0 | 112.8 | 279.9 | 77474.49 | 97144.94 |
| **D1** | 12.10 | 186.60 | 2.10 | ND | 7.40 | 9.60 | 0.20 | 111.40 | 49.20 | 33.70 | 0 | 107.2 | 280.9 | 76369.41 | 98557.97 |
| **D2** | 9.70 | 229.70 | 2.90 | ND | 7.60 | 9.00 | ND | 77.90 | 36.00 | 27.00 | 0 | 83 | 412.1 | 78740.44 | 85464.89 |
| **D3** | 2.50 | 2029.80 | 4.50 | 3.20 | 23.00 | 9.10 | ND | 29.30 | 12.60 | 12.20 | 0 | 31.9 | 34.7 | 33103.44 | 22968.46 |
| **E1** | 2.60 | 150.20 | 1.00 | 0.90 | 5.40 | 9.30 | ND | 52.80 | 17.40 | 18.90 | 0 | 38.2 | 569.3 | 64234.51 | 43872.93 |
| **E2** | 12.90 | 340.80 | 4.90 | 4.60 | 8.60 | 13.60 | ND | 101.80 | 42.40 | 28.00 | 0 | 102.1 | 293.9 | 74159.25 | 83718.44 |
| **E3** | 6.10 | 364.10 | 1.10 | 0.50 | 3.80 | 10.90 | ND | 32.10 | 11.30 | 7.00 | 0 | 19.3 | 132.8 | 41160.73 | 24190.97 |
| **F1** | 13.60 | 344.40 | 3.60 | 3.40 | 6.20 | 14.00 | 1.00 | 114.40 | 35.10 | 26.10 | 0 | 91.1 | 368 | 66011.04 | 75388.41 |
| **F2** | 9.90 | 488.25 | 1.80 | 2.90 | 10.05 | 11.20 | 0.50 | 91.55 | 34.90 | 23.30 | 0 | 85.9 | 270.35 | 68850.67 | 76706.18 |
| **F3** | 6.20 | 632.10 | ND | 2.40 | 13.90 | 8.40 | ND | 68.70 | 34.70 | 20.50 | 0 | 80.7 | 172.7 | 71690.31 | 78061 |
| **G1** | 11.30 | 319.00 | 0.60 | 6.80 | 4.10 | 12.10 | ND | 77.40 | 31.10 | 21.20 | 0 | 72.4 | 232 | 65738.26 | 76176.96 |
| **G2** | 12.90 | 226.20 | 4.50 | 2.90 | 4.50 | 11.90 | 0.20 | 106.70 | 46.40 | 28.70 | 0 | 107.2 | 262.9 | 90098.98 | 91371.07 |
| **G3** | 18.30 | 277.90 | 3.50 | 7.30 | 7.10 | 13.30 | 0.70 | 116.90 | 51.70 | 30.60 | 0 | 128.8 | 274.7 | 71711.29 | 98505.05 |
| **H1** | 8.53 | 722.65 | 1.03 | 2.25 | 12.53 | 9.40 | 0.08 | 79.55 | 32.38 | 23.83 | 0 | 74.55 | 324.1 | 69662.00 | 69513.99 |
| **H2** | 11.26 | 515.93 | 0.51 | 5.28 | 8.91 | 6.90 | 0.19 | 42.38 | 18.39 | 13.66 | 0 | 42.975 | 199.5 | 44162.10 | 42385.8 |
| **H3** | 14.00 | 309.20 | ND | 8.30 | 5.30 | 4.40 | 0.30 | 5.20 | 4.40 | 3.50 | 0 | 11.4 | 74.9 | 18569.54 | 15194.11 |

Table S3. The major elements concentration (%) in marine sediments.

|  | SiO_2_ | TiO_2_ | Al_2_O_3_ | Fe_2_O_3_ | MnO | CaO | MgO | K_2_O | Na_2_O | P_2_O_5_ | SO_3_ | Cl | LOI | SrO |
| --- | --- | --- | --- | --- | --- | --- | --- | --- | --- | --- | --- | --- | --- | --- |
| **A2** | 7.073 | 0.324 | 3.290 | 1.932 | 0.050 | 43.666 | 0.524 | 0.160 | 1.057 | 0.065 | 0.416 | 1.634 | 38.120 | 1.666 |
| **A3** | 5.658 | 0.901 | 2.100 | 1.444 | ND | 45.048 | 0.503 | 0.316 | 0.480 | 0.043 | 0.698 | 1.149 | 40.180 | 1.431 |
| **B1** | 48.576 | 1.758 | 13.728 | 9.810 | 0.210 | 7.884 | 1.930 | 1.475 | 1.834 | 0.296 | 0.272 | 1.471 | 10.350 | 0.149 |
| **B2** | 47.936 | 1.705 | 14.480 | 10.261 | 0.209 | 7.671 | 2.125 | 1.427 | 1.829 | 0.215 | 0.375 | 1.494 | 9.860 | 0.174 |
| **B3** | 45.095 | 1.488 | 18.066 | 10.365 | 0.241 | 5.492 | 1.823 | 1.478 | 1.448 | 0.251 | 0.464 | 1.077 | 12.300 | 0.116 |
| **C1** | 31.667 | 1.826 | 11.946 | 9.980 | 0.128 | 18.173 | 1.228 | 1.378 | 1.028 | 0.200 | 0.510 | 0.924 | 20.250 | 0.548 |
| **C2** | 47.225 | 1.831 | 16.983 | 10.683 | 0.220 | 4.803 | 1.822 | 1.286 | 1.452 | 0.269 | 0.524 | 1.207 | 11.480 | 0.075 |
| **C3** | 46.127 | 1.591 | 18.356 | 11.077 | 0.202 | 2.813 | 1.657 | 1.606 | 0.983 | 0.245 | 0.838 | 0.637 | 13.620 | 0.042 |
| **D1** | 47.624 | 1.777 | 18.623 | 10.919 | 0.322 | 2.791 | 1.674 | 1.593 | 1.091 | 0.262 | 0.751 | 0.612 | 11.640 | 0.041 |
| **D2** | 49.416 | 1.878 | 16.149 | 11.258 | 0.321 | 3.698 | 1.557 | 1.852 | 1.419 | ND | 0.976 | 1.368 | 9.830 | 0.059 |
| **D3** | 9.531 | 0.751 | 4.340 | 4.733 | 0.113 | 37.015 | 1.321 | 0.350 | 0.457 | 0.155 | 0.521 | 0.674 | 39.450 | 0.574 |
| **E1** | 55.286 | 3.345 | 8.290 | 9.184 | 0.460 | 5.373 | 1.486 | 1.189 | 1.867 | 0.316 | 0.371 | 3.474 | 8.850 | 0.065 |
| **E2** | 47.669 | 1.918 | 15.819 | 10.603 | 0.214 | 4.283 | 1.794 | 1.327 | 1.431 | 0.251 | 0.451 | 1.168 | 12.870 | 0.065 |
| **E3** | 50.469 | 0.610 | 4.571 | 5.885 | 0.154 | 17.532 | 0.902 | 0.759 | 2.010 | 0.159 | 0.585 | 3.761 | 12.350 | 0.162 |
| **F1** | 50.097 | 2.128 | 14.245 | 9.438 | 0.230 | 4.512 | 2.060 | 1.336 | 1.939 | 0.310 | 0.198 | 1.404 | 11.850 | 0.069 |
| **F2** | 43.800 | 1.829 | 14.494 | 9.844 | 0.180 | 7.401 | 1.707 | 1.502 | 1.570 | 0.271 | 0.450 | 1.470 | 15.163 | 0.110 |
| **F3** | 37.503 | 1.530 | 14.750 | 10.250 | 0.131 | 10.289 | 1.353 | 1.668 | 1.253 | 0.234 | 0.710 | 1.535 | 18.500 | 0.151 |
| **G1** | 51.251 | 1.520 | 14.394 | 9.399 | 0.223 | 4.276 | 1.952 | 1.599 | 2.249 | 0.345 | 0.404 | 1.796 | 10.420 | 0.077 |
| **G2** | 47.098 | 1.886 | 17.265 | 12.882 | 0.162 | 3.749 | 1.610 | 1.602 | 1.534 | 0.270 | 0.495 | 0.950 | 10.150 | 0.055 |
| **G3** | 47.204 | 1.846 | 18.613 | 10.253 | 0.130 | 3.733 | 1.827 | 1.582 | 1.442 | 0.252 | 0.418 | 0.968 | 11.500 | 0.045 |
| **H1** | 45.772 | 2.177 | 13.135 | 9.960 | 0.280 | 8.545 | 1.580 | 1.400 | 1.445 | 0.269 | 0.476 | 1.600 | 12.765 | 0.201 |
| **H2** | 42.699 | 1.311 | 8.009 | 6.314 | 0.280 | 16.307 | 1.108 | 1.140 | 1.842 | 0.187 | 0.734 | 4.027 | 15.626 | 0.252 |
| **H3** | 39.609 | 0.445 | 2.871 | 2.655 | ND | 24.058 | 0.637 | 0.880 | 2.269 | 0.105 | 1.001 | 6.433 | 18.540 | 0.304 |

Table S3. Continued.

|  | Rb_2_O | V_2_O_5_ | Y_2_O_3_ | ZnO | ZrO_2_ | BaO | Co_3_O_4_ | Cr_2_O_3_ | CuO | SUM |
| --- | --- | --- | --- | --- | --- | --- | --- | --- | --- | --- |
| **A2** | ND | ND | ND | ND | ND | ND | 0.022 | ND | ND | 99.999 |
| **A3** | ND | ND | ND | 0.012 | ND | ND | 0.021 | ND | ND | 99.984 |
| **B1** | 0.009 | 0.049 | ND | 0.024 | 0.085 | 0.091 | ND | ND | ND | 100.001 |
| **B2** | 0.010 | ND | 0.005 | 0.020 | 0.083 | 0.051 | ND | 0.049 | ND | 99.979 |
| **B3** | 0.010 | ND | 0.005 | 0.023 | 0.060 | 0.064 | 0.038 | 0.070 | ND | 99.974 |
| **C1** | 0.007 | 0.070 | ND | 0.022 | ND | 0.088 | ND | ND | 0.025 | 99.998 |
| **C2** | 0.009 | ND | 0.006 | 0.017 | 0.065 | ND | ND | 0.033 | ND | 99.990 |
| **C3** | 0.011 | 0.054 | 0.009 | 0.020 | 0.057 | ND | ND | 0.019 | ND | 99.964 |
| **D1** | 0.012 | ND | 0.010 | 0.026 | 0.067 | ND | ND | 0.031 | 0.045 | 99.911 |
| **D2** | 0.013 | ND | 0.011 | 0.018 | 0.132 | ND | 0.043 | ND | ND | 99.998 |
| **D3** | ND | ND | 0.006 | 0.009 | ND | ND | ND | ND | ND | 100.000 |
| **E1** | ND | ND | 0.009 | ND | 0.262 | ND | ND | 0.173 | ND | 100.000 |
| **E2** | 0.008 | ND | 0.008 | 0.021 | 0.070 | ND | ND | 0.031 | ND | 100.001 |
| **E3** | ND | ND | ND | 0.011 | 0.080 | ND | ND | ND | ND | 100.000 |
| **F1** | 0.008 | ND | ND | 0.020 | 0.083 | ND | ND | 0.074 | ND | 100.001 |
| **F2** | 0.011 | ND | 0.004 | 0.022 | 0.074 | ND | ND | 0.071 | 0.033 | 100.003 |
| **F3** | 0.014 | ND | 0.004 | 0.023 | 0.064 | ND | ND | ND | 0.038 | 100.000 |
| **G1** | 0.008 | ND | 0.005 | 0.015 | 0.066 | ND | ND | ND | ND | 99.999 |
| **G2** | 0.009 | 0.080 | 0.008 | 0.029 | 0.065 | ND | 0.021 | 0.049 | 0.018 | 99.987 |
| **G3** | ND | ND | 0.008 | 0.019 | 0.048 | 0.041 | 0.010 | ND | 0.021 | 99.960 |
| **H1** | 0.009 | ND | 0.010 | 0.024 | 0.138 | 0.086 | ND | 0.102 | 0.033 | 100.005 |
| **H2** | ND | ND | 0.010 | ND | 0.128 | ND | ND | ND | 0.031 | 100.004 |
| **H3** | ND | ND | ND | ND | 0.122 | ND | 0.072 | ND | ND | 100.001 |

ND: Not detected.

Table S4. PSA of the studied samples (%).

|  | **A2** | **A3** | **B1** | **B2** | **B3** | **C1** | **C2** | **C3** | **D1** | **D2** | **D3** |
| --- | --- | --- | --- | --- | --- | --- | --- | --- | --- | --- | --- |
| Silt | 4.95 | 76.95 | 34.17 | 40.06 | 79.85 | 67 | 63.4 | 79.31 | 58.92 | 73.52 | 65.78 |
| Clay | 0.51 | 8.52 | 4.83 | 5.07 | 11.72 | 10.64 | 7.14 | 11.74 | 11.32 | 10.55 | 12.50 |
| sand | 90.86 | 14.49 | 61.02 | 54.9 | 8.44 | 18.99 | 29.48 | 7.07 | 29.73 | 14.34 | 17.90 |
| gravel | 3.66 | 0.00 | 0.00 | 0.00 | 0.00 | 3.37 | 0.00 | 1.88 | 0.00 | 1.60 | 3.79 |
| sum | 99.98 | 99.96 | 100.02 | 100.03 | 100.01 | 100 | 100.02 | 100 | 99.97 | 100.01 | 99.97 |

Table S4. Continue.

|  | **E1** | **E2** | **E3** | **F1** | **F2** | **F3** | **G1** | **G2** | **G3** | **H1** | **H2** | **H3** |
| --- | --- | --- | --- | --- | --- | --- | --- | --- | --- | --- | --- | --- |
| Silt | 3.93 | 46.02 | 2.95 | 27.34 | 42.11 | 32.27 | 33.08 | 84.08 | 85.88 | 55.18 | 77.50 | 73.91 |
| Clay | 0.58 | 7.06 | 0.27 | 1.80 | 4.02 | 6.05 | 2.60 | 9.71 | 10.57 | 5.67 | 8.01 | 7.14 |
| sand | 95.49 | 35.5 | 91.26 | 65.34 | 40.27 | 45.97 | 64.35 | 6.25 | 3.56 | 39.13 | 11.86 | 17.63 |
| gravel | 0.00 | 11.42 | 5.46 | 5.50 | 13.54 | 15.73 | 0.00 | 0.00 | 0.00 | 0.00 | 2.62 | 1.29 |
| sum | 100 | 100 | 99.94 | 99.98 | 99.94 | 100.02 | 100.03 | 100.04 | 100.01 | 99.98 | 99.99 | 99.97 |

Wt% clay: <2 μm; wt% silt: 2 to 63 μm; wt% sand: 2–0.063 mm; wt% gravel: >2 mm. The PSA analysis was repeated six times for each sample, and only the average results with STD ±2% were reported in this table.

Table S5. Distribution of the main minerals in the study area according to FTIR spectra

|  | **Quartz** | **Microcline** | **Albite** | **Kaolinite** | **Montmorlinite** | **Calcite** | **Aragonite** |
| --- | --- | --- | --- | --- | --- | --- | --- |
| **A2** |  |  |  | Kaolinite | Montmorlinite | Calcite | Aragonite |
| **A3** |  |  |  | Kaolinite | Montmorlinite | Calcite | Aragonite |
| **B1** | Quartz | Microcline |  | Kaolinite | Montmorlinite |  | Aragonite |
| **B2** | Quartz | Microcline | Albite | Kaolinite | Montmorlinite | Calcite | Aragonite |
| **B3** | Quartz | Microcline | Albite | Kaolinite | Montmorlinite | Calcite | Aragonite |
| **C1** | Quartz | Microcline | Albite | Kaolinite | Montmorlinite | Calcite |  |
| **C2** | Quartz | Microcline | Albite | Kaolinite | Montmorlinite | Calcite |  |
| **C3** | Quartz |  | Albite | Kaolinite | Montmorlinite | Calcite |  |
| **D1** | Quartz |  | Albite | Kaolinite | Montmorlinite | Calcite |  |
| **D2** | Quartz |  | Albite | Kaolinite | Montmorlinite | Calcite |  |
| **D3** | Quartz | Microcline | Albite | Kaolinite | Montmorlinite | Calcite | Aragonite |
| **E1** | Quartz | Microcline |  | Kaolinite | Montmorlinite |  |  |
| **E2** | Quartz | Microcline | Albite | Kaolinite | Montmorlinite | Calcite |  |
| **E3** | Quartz | Microcline |  | Kaolinite |  | Calcite | Aragonite |
| **F1** | Quartz | Microcline | Albite |  | Montmorlinite |  |  |
| **F2** | Quartz | Microcline | Albite | Kaolinite | Montmorlinite | Calcite |  |
| **F3** | Quartz | Microcline | Albite | Kaolinite | Montmorlinite | Calcite |  |
| **G1** | Quartz | Microcline | Albite |  | Montmorlinite | Calcite |  |
| **G2** | Quartz |  | Albite | Kaolinite | Montmorlinite | Calcite |  |
| **G3** | Quartz |  |  | Kaolinite | Montmorlinite | Calcite |  |
| **H1** | Quartz |  | Albite | Kaolinite | Montmorlinite | Calcite |  |
| **H2** | Quartz | Microcline | Albite | Kaolinite | Montmorlinite | Calcite |  |
| **H3** | Quartz | Microcline |  | Kaolinite | Montmorlinite | Calcite |  |

Table S6. Distribution of the main minerals % in the study area according to XRD.

|  | **A2** | **A3** | **B1** | **B2** | **B3** | **C1** | **C2** | **C3** | **D1** | **D2** | **D3** | **E1** |
| --- | --- | --- | --- | --- | --- | --- | --- | --- | --- | --- | --- | --- |
| Aragonite | 71.8 | 77.2 |  | 4.9 | 35.5 |  |  |  |  |  | 32.9 | 6.8 |
| Calcite | 20.2 | 13.9 |  |  | 5.2 |  |  |  |  |  | 22.9 |  |
| Quartz |  | 6.7 | 44.1 | 47.5 | 18.9 | 51.9 | 15.4 | 15.4 |  | 60.9 | 7.5 | 78.4 |
| Albite |  |  | 33.2 |  |  | 48.1 |  |  |  | 21.7 | 37.1 |  |
| Muscovite |  |  |  | 3.7 | 40.3 |  |  |  |  |  |  | 14.8 |
| Illite |  |  |  |  |  |  | 26.4 | 26.4 |  |  |  |  |
| Kaolinite |  |  |  |  |  |  | 4.4 | 4.5 |  |  |  |  |
| Berlinite |  |  |  |  |  |  |  |  | 84.2 |  |  |  |
| Microcline |  |  |  |  |  |  | 12.1 | 12.1 |  | 16.7 |  |  |
| Montmorillonite |  |  |  |  |  |  |  |  |  | 0.7 |  |  |

Table S6. Continue

|  | **E2** | **E3** | **F1** | **F2** | **F3** | **G1** | **G2** | **G3** | **H1** | **H2** | **H3** |
| --- | --- | --- | --- | --- | --- | --- | --- | --- | --- | --- | --- |
| Aragonite |  |  |  |  | 30.8 |  |  |  |  |  |  |
| Calcite |  |  |  |  | 33 |  |  |  |  |  |  |
| Quartz | 17.7 | 91.4 |  |  |  | 45.7 |  | 57 | 30.4 | 55 | 57.8 |
| Albite |  |  | 33.6 | 43.6 |  |  | 24.7 | 42.1 | 44.4 | 42.5 | 42.2 |
| Muscovite | 37.8 |  |  |  |  |  |  |  |  |  |  |
| Illite |  |  |  |  |  |  |  |  |  |  |  |
| Kaolinite |  |  |  |  |  |  |  |  |  |  |  |
| Berlinite |  |  | 41.8 | 36.8 | 36.2 |  | 47.6 |  |  |  |  |
| Microcline | 30.2 |  | 24.5 |  |  |  | 27.7 |  | 25.2 |  |  |
| Montmorillonite |  |  |  |  |  | 1.4 |  | 0.9 |  |  |  |

Table S7. The *PLI* and *C*_d_ values were calculated in the collected sediment samples.

| **Location** | ***CD*** | ***PLI*** |
| --- | --- | --- |
| **A2** | 7.19 | 0.24 |
| **A3** | 10.46 | 0.23 |
| **B1** | 16.94 | 0.85 |
| **B2** | 15.85 | 0.87 |
| **B3** | 16.94 | 1.00 |
| **C1** | 9.47 | 0.62 |
| **C2** | 16.33 | 0.96 |
| **C3** | 16.39 | 0.95 |
| **D1** | 18.63 | 1.00 |
| **D2** | 13.72 | 0.79 |
| **D3** | 18.49 | 0.34 |
| **E1** | 13.44 | 0.55 |
| **E2** | 20.89 | 1.01 |
| **E3** | 10.20 | 0.27 |
| **F1** | 16.48 | 0.96 |
| **F2** | 15.11 | 0.85 |
| **F3** | 13.73 | 0.71 |
| **G1** | 12.82 | 0.70 |
| **G2** | 23.10 | 0.95 |
| **G3** | 20.41 | 1.09 |
| **H1** | 14.62 | 0.78 |
| **H2** | 12.24 | 0.51 |
| **H3** | 9.86 | 0.18 |

Table S8. The heavy metal concentrations (mg kg^–1^) in shales were determined by several criteria and compared with sediment quality recommendations (*SQGs*).

| Metals | Shale | This study (average concentrations) | SQG | | | TEL | PEL | ERL | ERM |
| --- | --- | --- | --- | --- | --- | --- | --- | --- | --- |
|  |  |  | **Non-polluted** | **Moderate polluted** | **Heavily polluted** |  |  |  |  |
| Cu | 45 | 9.78 | >25 | 25-50 | >50 | 18.7 | 110 | 34 | 270 |
| Cd | 0.30 | 2.44 | - | - | - | 0.68 | 4.2 | 1.2 | 9.6 |
| Zn | 95 | 74.23 | >90 | 90-200 | >200 | 124 | 270 | 150 | 410 |
| Co | 19 | 5.97 | - | - | - | - | - | - | - |
| Ni | 68 | 36.46 | >20 | 20-50 | >50 | 15.9 | 43 | 20.9 | 51.6 |
| Mn | 850 | 509.36 | - | - | - | - | - | - | - |
| Fe | 47600 | 60554 | - | - | - | - | - | - | - |
| Al | 80000 | 65467 | - | - | - | - | - | - | - |

Table S9. Correlation analysis of the studied matrix.


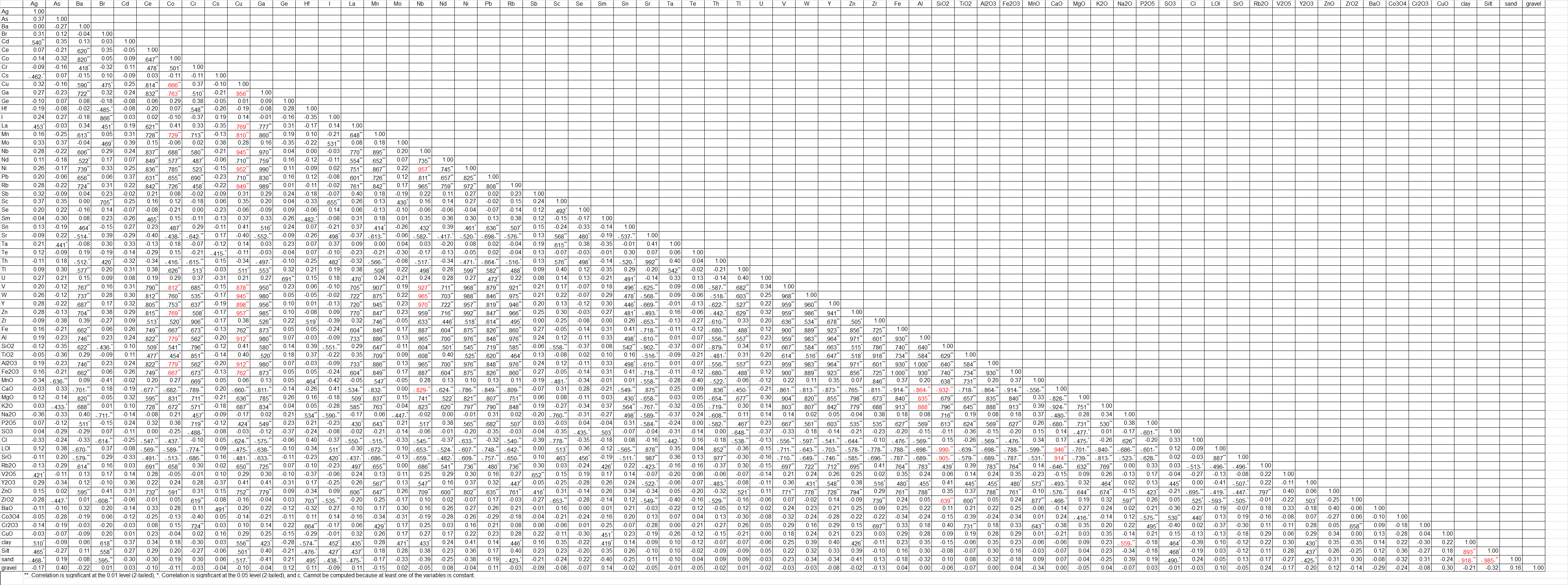


Table S10. Descriptive Statistics of the studied Trace elements.

|  | N | Minimum | Maximum | Mean | Std. Deviation |
| --- | --- | --- | --- | --- | --- |
| Ag | 23 | 0.600 | 1.700 | 1.067 | 0.315 |
| As | 23 | 7.400 | 29.300 | 15.156 | 5.821 |
| Ba | 23 | 0.000 | 136.400 | 60.377 | 42.210 |
| Br | 23 | 16.700 | 79.700 | 40.871 | 17.085 |
| Cd | 23 | 1.000 | 4.300 | 2.443 | 0.796 |
| Ce | 23 | 0.000 | 65.400 | 35.040 | 17.799 |
| Co | 23 | 0.100 | 13.200 | 5.970 | 3.807 |
| Cr | 23 | 7.700 | 142.700 | 66.817 | 33.522 |
| Cs | 23 | 0.000 | 12.200 | 5.752 | 3.303 |
| Cu | 23 | 3.600 | 16.500 | 9.777 | 4.004 |
| Ga | 23 | 3.400 | 27.600 | 15.712 | 7.944 |
| Ge | 23 | 0.000 | 0.600 | 0.048 | 0.141 |
| Hf | 23 | 1.200 | 14.500 | 6.792 | 2.548 |
| Hg | 23 | 0.000 | 0.000 | 0.000 | 0.000 |
| I | 23 | 0.000 | 75.900 | 17.759 | 23.002 |
| La | 23 | 0.700 | 34.300 | 16.930 | 10.652 |
| Mn | 23 | 47.200 | 1043.000 | 509.356 | 256.805 |
| Mo | 23 | 0.000 | 1.900 | 0.883 | 0.491 |
| Nb | 23 | 0.000 | 28.000 | 14.313 | 8.898 |
| Nd | 23 | 3.000 | 31.400 | 15.615 | 6.176 |
| Ni | 23 | 1.000 | 64.400 | 36.465 | 20.134 |
| Pb | 23 | 0.000 | 13.800 | 6.534 | 3.327 |
| Rb | 23 | 0.000 | 64.500 | 31.209 | 19.423 |
| Sb | 23 | 0.000 | 11.600 | 2.739 | 2.955 |
| Sc | 23 | 4.800 | 29.000 | 17.988 | 5.703 |
| Se | 23 | 0.000 | 3.700 | 1.596 | 0.821 |
| Sm | 23 | 0.000 | 19.000 | 6.989 | 5.929 |
| Sn | 23 | 0.000 | 18.300 | 8.934 | 4.520 |
| Sr | 23 | 150.200 | 5592.700 | 995.584 | 1523.087 |
| Ta | 23 | 0.000 | 4.900 | 2.1896 | 1.707 |
| Te | 23 | 0.000 | 12.900 | 3.736 | 3.294 |
| Th | 23 | 3.800 | 79.100 | 15.652 | 20.252 |
| Tl | 23 | 4.400 | 14.600 | 10.326 | 2.423 |
| U | 23 | 0.000 | 1.000 | 0.151 | 0.259 |
| V | 23 | 4.400 | 116.900 | 74.242 | 38.131 |
| W | 23 | 2.500 | 51.700 | 31.190 | 15.639 |
| Y | 23 | 0.500 | 36.800 | 21.078 | 10.374 |
| Yb | 23 | 0.000 | 0.000 | 0.000 | 0.000 |
| Zn | 23 | 11.400 | 128.800 | 74.232 | 35.444 |
| Zr | 23 | 0.000 | 569.300 | 237.472 | 129.881 |
| Fe | 23 | 10099.591 | 90098.978 | 60554.095 | 22294.163 |
| Al | 23 | 11113.770 | 98557.972 | 65467.009 | 29460.470 |
| Valid N (listwise) | 23 |  |  |  |  |

Table S11. Descriptive Statistics of the studied major elements.

|  | N | Minimum | Maximum | Mean | Std. Deviation |
| --- | --- | --- | --- | --- | --- |
| SiO_2_ | 23 | 5.658 | 55.286 | 41.060 | 14.188 |
| TiO_2_ | 23 | 0.324 | 3.345 | 1.581 | 0.655 |
| Al_2_O_3_ | 23 | 2.100 | 18.623 | 12.370 | 5.567 |
| Fe_2_O_3_ | 23 | 1.444 | 12.882 | 8.658 | 3.188 |
| MnO | 21 | 0.050 | 0.460 | 0.212 | 0.089 |
| CaO | 23 | 2.791 | 45.048 | 12.570 | 12.978 |
| MgO | 23 | 0.503 | 2.125 | 1.486 | 0.474 |
| K_2_O | 23 | 0.160 | 1.852 | 1.257 | 0.460 |
| Na_2_O | 23 | 0.457 | 2.269 | 1.475 | 0.483 |
| P_2_O_5_ | 23 | 0.000 | 0.345 | 0.216 | 0.090 |
| SO_3_ | 23 | 0.198 | 1.001 | 0.549 | 0.208 |
| Cl | 23 | 0.612 | 6.433 | 1.775 | 1.378 |
| LOI | 23 | 8.850 | 40.180 | 16.333 | 9.551 |
| SrO | 23 | 0.041 | 1.666 | 0.280 | 0.427 |
| Rb_2_O | 23 | 0.000 | 0.014 | 0.006 | 0.005 |
| V_2_O_5_ | 23 | 0.000 | 0.080 | 0.011 | 0.025 |
| Y_2_O_3_ | 22 | 0.000 | 0.011 | 0.005 | 0.004 |
| ZnO | 23 | 0.000 | 0.029 | 0.016 | 0.009 |
| ZrO_2_ | 23 | 0.000 | 0.262 | 0.076 | 0.057 |
| BaO | 23 | 0.000 | 0.091 | 0.018 | 0.033 |
| Co_3_O_4_ | 23 | 0.000 | 0.072 | 0.010 | 0.019 |
| Cr_2_O_3_ | 23 | 0.000 | 0.173 | 0.031 | 0.044 |
| CuO | 23 | 0.000 | 0.045 | 0.011 | 0.016 |
| Valid N (listwise) | 21 |  |  |  |  |

Table S12. The principal component analysis of trace element.

| Component Matrix^a^ | | | | | | | | |
| --- | --- | --- | --- | --- | --- | --- | --- | --- |
|  | Component | | | | | | | |
|  | 1 | 2 | 3 | 4 | 5 | 6 | 7 | 8 |
| Ag | .231 | .608 | .215 | -.487 | -.251 | .125 | -.267 | .132 |
| As | -.213 | .416 | .460 | -.134 | .026 | -.202 | -.004 | -.322 |
| Ba | .739 | -.139 | -.017 | -.005 | -.189 | .344 | .388 | -.074 |
| Br | .195 | .851 | -.257 | .060 | .045 | -.194 | .073 | .264 |
| Cd | .242 | .336 | .457 | -.501 | -.253 | -.182 | -.232 | -.156 |
| Ce | .829 | .101 | -.346 | .153 | .237 | .007 | .080 | .028 |
| Co | .787 | -.107 | .093 | .180 | -.008 | .224 | .402 | .061 |
| Cr | .650 | -.425 | .353 | -.027 | .401 | .072 | -.113 | .092 |
| Cs | -.189 | .102 | -.211 | -.223 | .489 | -.238 | .550 | -.336 |
| Cu | .897 | .332 | -.175 | .025 | -.036 | -.043 | -.032 | -.004 |
| Ga | .977 | .141 | -.094 | .040 | -.092 | .029 | .002 | .002 |
| Ge | .214 | -.259 | .442 | .657 | .188 | -.343 | .077 | .010 |
| Hf | -.022 | -.526 | .572 | -.135 | .191 | .204 | -.161 | .329 |
| I | -.102 | .801 | -.012 | -.098 | .049 | -.195 | .256 | .411 |
| La | .743 | .244 | -.011 | .290 | -.067 | -.377 | -.264 | .095 |
| Mn | .912 | -.072 | .049 | -.050 | .158 | .054 | -.104 | -.030 |
| Mo | .084 | .624 | .035 | -.565 | .108 | -.211 | .022 | -.015 |
| Nb | .966 | .096 | -.095 | -.042 | .053 | .006 | -.144 | .049 |
| Nd | .756 | -.006 | -.228 | .114 | .056 | -.032 | .026 | .131 |
| Ni | .976 | .173 | -.065 | .024 | -.024 | .056 | .037 | -.030 |
| Pb | .879 | -.082 | .180 | -.122 | -.105 | -.074 | .047 | .069 |
| Rb | .964 | .101 | -.169 | -.018 | -.109 | .018 | .000 | .025 |
| Sb | .181 | .268 | -.008 | .514 | -.182 | -.014 | -.252 | -.397 |
| Sc | .112 | .901 | .162 | .107 | .122 | .177 | .009 | .046 |
| Se | -.060 | .507 | .146 | .408 | .155 | .355 | -.307 | -.188 |
| Sm | .288 | -.009 | -.786 | .153 | -.091 | -.104 | -.224 | -.033 |
| Sn | .511 | -.251 | .230 | .015 | -.535 | -.259 | .274 | -.048 |
| Sr | -.656 | .595 | -.022 | .340 | .081 | .225 | .062 | .084 |
| Ta | -.011 | .608 | .604 | .140 | .035 | .085 | -.004 | -.150 |
| Te | -.081 | -.115 | .097 | .114 | -.791 | .359 | .146 | .086 |
| Th | -.605 | .597 | -.060 | .367 | .103 | .236 | .026 | .121 |
| Tl | .563 | .265 | .576 | .051 | .163 | .183 | .330 | -.046 |
| U | .314 | -.129 | .450 | .492 | -.188 | -.526 | .016 | .172 |
| V | .984 | .026 | .107 | .003 | .044 | .041 | .057 | -.063 |
| W | .977 | .130 | -.031 | -.055 | -.033 | .063 | .020 | -.055 |
| Y | .980 | .007 | -.038 | -.083 | .050 | .039 | -.090 | -.035 |
| Zn | .969 | .222 | -.010 | .018 | -.037 | .022 | .041 | .014 |
| Zr | .653 | -.459 | .177 | -.066 | .403 | .126 | -.213 | .157 |
| Fe | .916 | -.126 | -.063 | -.096 | .102 | .061 | -.097 | -.192 |
| Al | .982 | .034 | -.083 | .010 | -.024 | .062 | .005 | -.044 |
| Total | 17.532 | 5.918 | 3.209 | 2.529 | 2.028 | 1.577 | 1.455 | 1.014 |
| % of variance | 43.831 | 14.795 | 8.023 | 6.323 | 5.070 | 3.943 | 3.637 | 2.535 |
| % of cumulative | 43.831 | 58.625 | 66.649 | 72.971 | 78.041 | 81.984 | 85.621 | 88.156 |

a. 8 components extracted.

Table S13. The Principle component analysis of major elements.

| Component Matrix^a^ | | | | | | |
| --- | --- | --- | --- | --- | --- | --- |
|  | Component | | | | | |
|  | 1 | 2 | 3 | 4 | 5 | 6 |
| SiO_2_ | .891 | .294 | -.017 | -.283 | -.105 | .061 |
| TiO_2_ | .780 | .304 | -.050 | .389 | .193 | .096 |
| Al_2_O_3_ | .813 | -.493 | .082 | -.040 | .057 | -.140 |
| Fe_2_O_3_ | .921 | -.328 | .057 | .030 | .043 | .062 |
| MnO | .607 | .590 | .316 | .187 | .179 | .043 |
| CaO | -.981 | .008 | -.041 | .130 | .046 | .051 |
| MgO | .798 | -.161 | -.308 | -.044 | .167 | -.356 |
| K_2_O | .901 | -.272 | .153 | -.112 | -.084 | .121 |
| Na_2_O | .432 | .579 | -.325 | -.522 | -.117 | .088 |
| P_2_O_5_ | .599 | .134 | -.588 | .262 | -.236 | -.187 |
| SO_3_ | .012 | -.215 | .905 | -.095 | -.227 | .105 |
| Cl | -.142 | .860 | .071 | -.243 | -.279 | .273 |
| LOI | -.911 | -.197 | .018 | .285 | .064 | -.139 |
| SrO | -.884 | -.072 | -.068 | .119 | .202 | .127 |
| Rb_2_O | .611 | -.550 | .138 | -.047 | .028 | -.045 |
| V_2_O_5_ | .122 | -.456 | -.202 | .052 | -.002 | .568 |
| Y_2_O_3_ | .421 | .119 | .705 | .307 | .031 | -.139 |
| ZnO | .612 | -.703 | -.163 | .014 | -.037 | .060 |
| ZrO_2_ | .497 | .776 | .223 | .124 | .123 | .166 |
| BaO | .154 | -.246 | -.442 | .144 | .216 | .586 |
| Co_3_O_4_ | -.043 | -.211 | .444 | -.331 | .673 | .179 |
| Cr_2_O_3_ | .430 | .528 | -.134 | .500 | .357 | -.006 |
| CuO | .159 | -.207 | .235 | .469 | -.610 | .253 |
| Total | 9.291 | 4.209 | 2.611 | 1.520 | 1.384 | 1.161 |
| % of variance | 40.395 | 18.300 | 11.350 | 6.609 | 6.016 | 5.047 |
| % of cumulative | 40.395 | 58.694 | 70.045 | 76.654 | 82.670 | 87.717 |

^a.^ 6 components extracted.

Table S14. Comparison between trace metals concentrations (μg/g dry weight) in the present study and other studies from Egypt and other countries.

| **Study area** | **Cr** | **Co** | **Ni** | **Zn** | **Cd** | **Pb** | **Ref.** |
| --- | --- | --- | --- | --- | --- | --- | --- |
| Nile Delta coastal sediments | 7.7 -142.7 | 0.1-13.2 | 1.0-64.4 | 11.4-128.8 | 1.0-4.3 | ND-13.8 | Present Study |
| Nile Delta coastal sediments | 209.9 | 22.3 | 45.2 | 68.9 | 0.127 | 8.77 | [1] |
| Nile Delta coastal sediments | 64.0 - 109.6 | 42.78 –30.9 | 70.05 –2.5 | 70.03 –35.9 | 7.16 –3.07 | 69.9 –49.9 | [2] |
| Nile Delta coastal sediments | 84.7 | 6.91 | 8.64 | 6.81 | 0.07 | 1.37 | [3] |
| Nile Delta coastal sediments | 102.3 | 23.24 | 86.31 | 59.11 | 4.09 | 2.58 | [4] |
| Edku lagoon, Egypt | 108.34 | 29.46 | 59.22 | 0.65 | ND | 64.0 | [5] |
| Burullus lagoon, Egypt | 103.0 | ND | 41.0 | 121.0 | 1.48 | 15.0 | [6] |
| Manzala lagoon, Egypt | 10.46 –44.22 | 4.18 –7.04 | 12.34 –18.49 | 25.8 –200 | ND -0.26 | 5.17 –285 | [7] |
| Yangtze River Delta, China | 78.9 | ND | ND | 94.3 | 0.26 | 31.8 | [8] |
| Simenit Lake | 154.0 | ND | 130.64 | 75.33 | 0.18 | 20.39 | [9] |
| Almus Dam Lake, Türkiye | 108.0 | ND | 72.0 | 86 | 0.16 | 9.34 | [10] |
| Abdal River, Türkiye | 96.28 | ND | 62.45 | 70.97 | 0.26 | 13.88 | [11] |

ND = not detected

**References**

1. Mandour, A., El-Sayed, M. K., El-Gamal, A. A., Khadr, A. M., & Elshazly, A. Temporal distribution of trace metals pollution load index in the Nile Delta coastal surface sediments. *Mar. Poll. Bull.*, **167**, 112290 https://doi.org/10.1016/j.marpolbul.2021.112290 (2021).‏
2. El Nemr, A.M., El Sikaily, & A., Khaled, A. Total and leachable heavy metals in muddy and sandy sediments of Egyptian coast along Mediterranean Sea. *Environ. Monit. Assess.* **129**, 151–168. <https://doi.org/10.1007/s10661-006-9349-8> (2007).
3. Okbah, M.A., Nasr, S.M., Soliman, N.F., & Khairy, M.A. Distribution and Contamination Status of Trace Metals in the Mediterranean Coastal Sediments, Egypt. *Soil. Sed. Contam.* **23**, 656–676. <https://doi.org/10.1080/15320383.2014.851644> (2014).
4. El-Gamal, A.A. & Saleh, I.H. Geochemical Assessment of Heavy Metals Pollution and Ecological Risk in the Nile Delta Coastal Sediments, Egypt. *JKAU: Mar. Sci.,* ***26*(1)**, 41-59. https://doi.org/10.4197/Mar.26-1.5 (2016).
5. Hamed, M.A., Mohamedein, L.I., El-Sawy, M.A., & El-Moselhy, K.M., 2013. Mercury and tin contents in water and sediments along the Mediterranean shoreline of Egypt. Egypt. J. Aquat. Res. 39, 75–81. <https://doi.org/10.1016/j.ejar.2013.06.001> (2013).
6. Gu, J., Salem, A., & Chen, Z. Lagoons of the Nile delta, Egypt, heavy metal sink: with a special reference to the Yangtze estuary of China. *Estuarine, Coastal and Shelf Science*, **117**, 282-292 (2013).‏
7. Elkady, A.A., Sweet, S.T., Wade, T.L., & Klein, A.G. Distribution and assessment of heavy metals in the aquatic environment of Lake Manzala. Egypt. Ecol. Indic. 58, 445–457. <https://doi.org/10.1016/j.ecolind.2015.05.029> (2015).
8. Zhang, W., Feng, H., Chang, J., Qu, J., Xie, H., & Yu, L. Heavy metal contamination in surface sediments of Yangtze River intertidal zone: an assessment from different indexes. *Environ. Pollut.* **157**, 1533–1543. [https://doi.org/10.1016/j. envpol.2009.01.007](https://doi.org/10.1016/j.%20envpol.2009.01.007) (2009).
9. Yüksel, B. & Ustaoğlu, F. Pollution analysis of metals in the sediments of lagoon lakes in Türkiye: Toxicological risk assessment and source insights. *Process* Safety *and Environmental Protection*, **193**, 665-682. https://doi.org/10.1016/j.psep.2024.11.085 (2025).
10. Yüksel, B., Ustaoğlu, F., Aydın, H., Tokatlı, C., Topaldemir, H., Islam, M.S. & Muhammad, S. Appraisal of metallic accumulation in the surface sediment of a fish breeding dam in Türkiye: A stochastical approach to ecotoxicological risk assessment. *Mar. Poll. Bull.*, **203**, 116488. https://doi.org/10.1016/j.marpolbul.2024.116488 (2024).
11. Ustaoğlu, F., Yüksel, B., Tepe, Y., Aydın, H. & Topaldemir, H. Metal pollution assessment in the surface sediments of a river system in Türkiye: Integrating toxicological risk assessment and source identification. *Mar. Poll. Bull.*, **203**, 116514. https://doi.org/10.1016/j.marpolbul.2024.116514 (2024).
